# Supplementary material for: Perturbed Redox Signaling Exacerbates a Mitochondrial Myopathy
Source: Cell Metab. 2018 Nov 6;28(5):764–775.e5. doi: 10.1016/j.cmet.2018.07.012 (PMC6224544; doi:10.1016/j.cmet.2018.07.012)
Supplement: Document S1. Figures S1–S5 and Table S1 [file mmc1.pdf]

Cell Metabolism, Volume *28*

Supplemental Information

Perturbed Redox Signaling

Exacerbates a Mitochondrial Myopathy

Sukru Anil Dogan, Raffaele Cerutti, Cristiane Benincá, Gloria Brea-Calvo, Howard Trevor Jacobs, Massimo Zeviani, Marten Szibor, and Carlo Viscomi

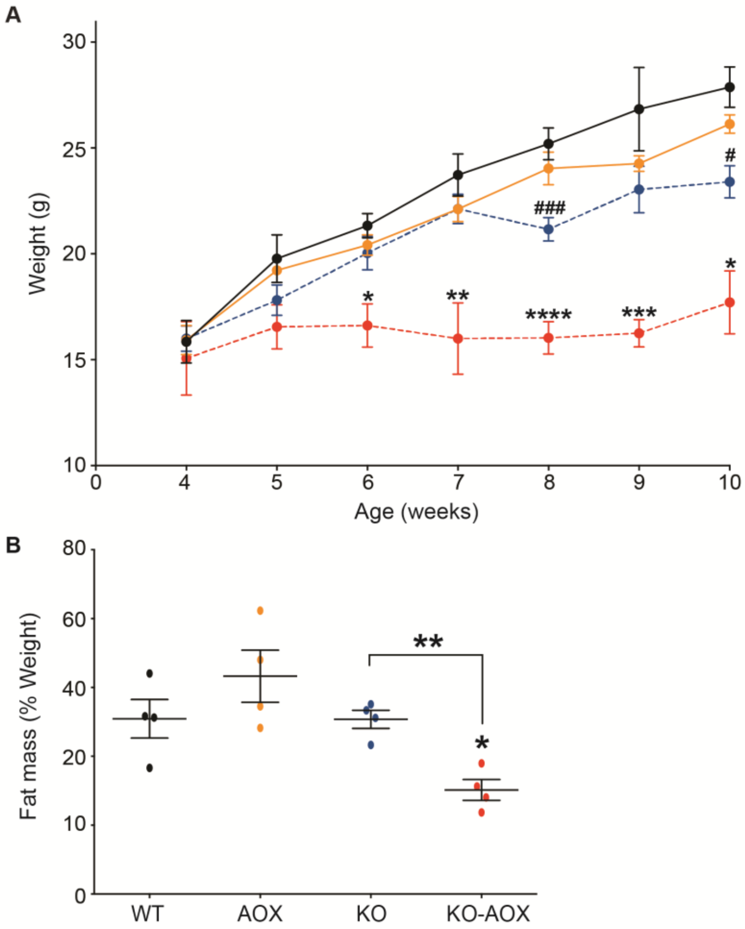

A
30
25
20
15
10
Weight (g)
0
4
5
6
7
8
9
10
Age (weeks)
###
#
*
*
**
****
***
B
80
60
40
20
10
0
Fat mass (% Weight)
**
*
WT
AOX
KO
KO-AOX

Figure S1. Related to Figure 1. KO-AOX is phenotypically worse than KO and control mice.

(A) Weight curve for male WT, AOX, KO and KO-AOX mice. Asterisks are used to compare the weight between KO and KO-AOX; the hash keys between WT and KO (n = 13-19). **(B)** Percentage of body fat in 8-week-old animals measured by NMR (n = 4).

Bars represent mean ± S.E.M. Asterisks and hash keys over the bars indicate statistical significance vs. WT; over the brackets among indicated groups. (*p, #p $\leq$ 0.05; **p $\leq$ 0.01; ***p, ###p $\leq$ 0.001; ****p < 0.0001; unpaired Student's *t*-test).

A

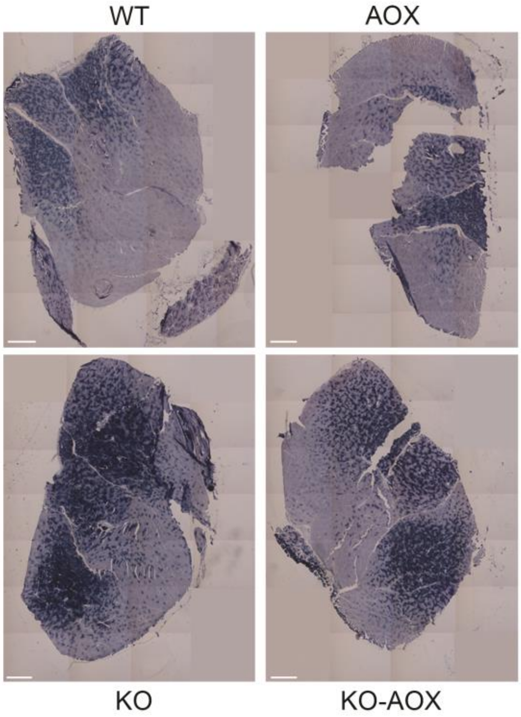
WT
AOX
KO
KO-AOX

B

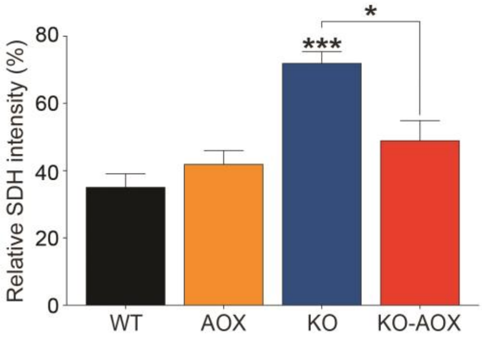
Relative SDH intensity (%)
80
60
40
20
0
***
*
WT
AOX
KO
KO-AOX

Figure S2. Related to Figure 2. SDH activity is increased in KO muscles but returns to WT levels in KO-AOX.

(A) Representative gastrocnemius muscle SDH activity micrograph. Whole muscle fiber was stained for SDH activity in 8-week-old WT, AOX, KO and KO-AOX animals (blue). The image represents a randomly chosen image from four samples. Scale bar: 500 μm. **(B)** Quantification of relative SDH intensity (% of total area) (n = 4).

Bars represent mean ± S.E.M. Asterisks over the bars indicate statistical significance vs. WT; over the brackets among indicated groups. (*p ≤ 0.05; ***p ≤ 0.001; unpaired Student's *t*-test).

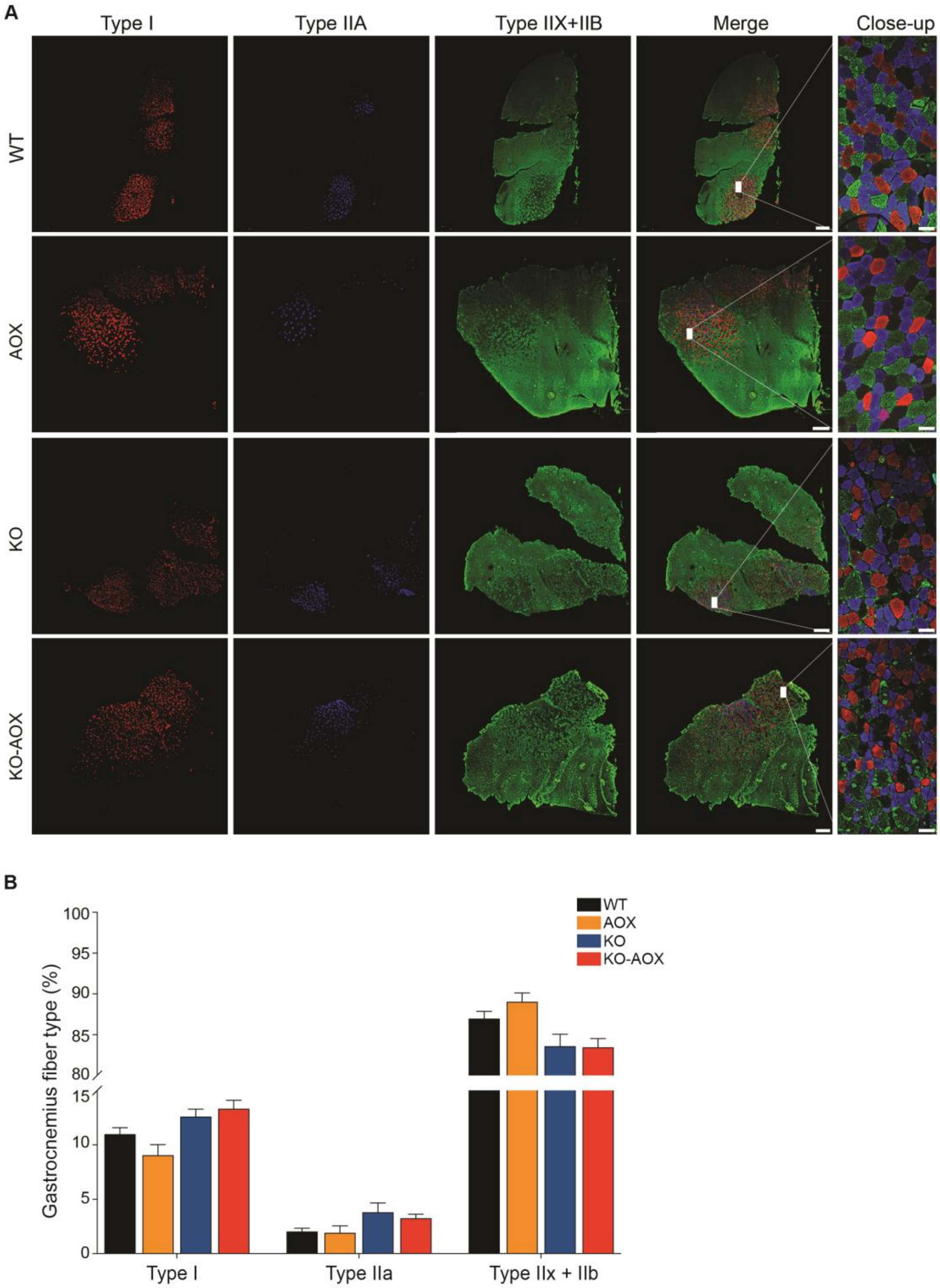

A
Type I
Type IIA
Type IIX+IIB
Merge
Close-up
WT
AOX
KO
KO-AOX
B
WT
AOX
KO
KO-AOX
Gastrocnemius fiber type (%)
100
95
90
85
80
15
10
5
0
Type I
Type IIa
Type IIx + IIb

Figure S3. Related to Figure 3. No differences were observed in the distribution of muscle fiber types among WT, AOX, KO and KO-AOX animals.

(A) Sections of 8-week-old gastrocnemius muscle were immunostained for the different MHCI, MHCIIA and MHCIIB myosins. Type I (Red), type IIA (Blue), type IIB (Green) and type IIX (unstained) fibers are shown in a whole tissue 3D montage. Scale bars: WT and KO 500 µm, AOX 400 µm and KO-AOX 300 µm. White rectangle highlights the zoomed area (scale bar: 50 µm). **(B)** Quantification of percentage of each fiber per whole tissue section (n = 4).

Bars represent mean ± S.E.M.

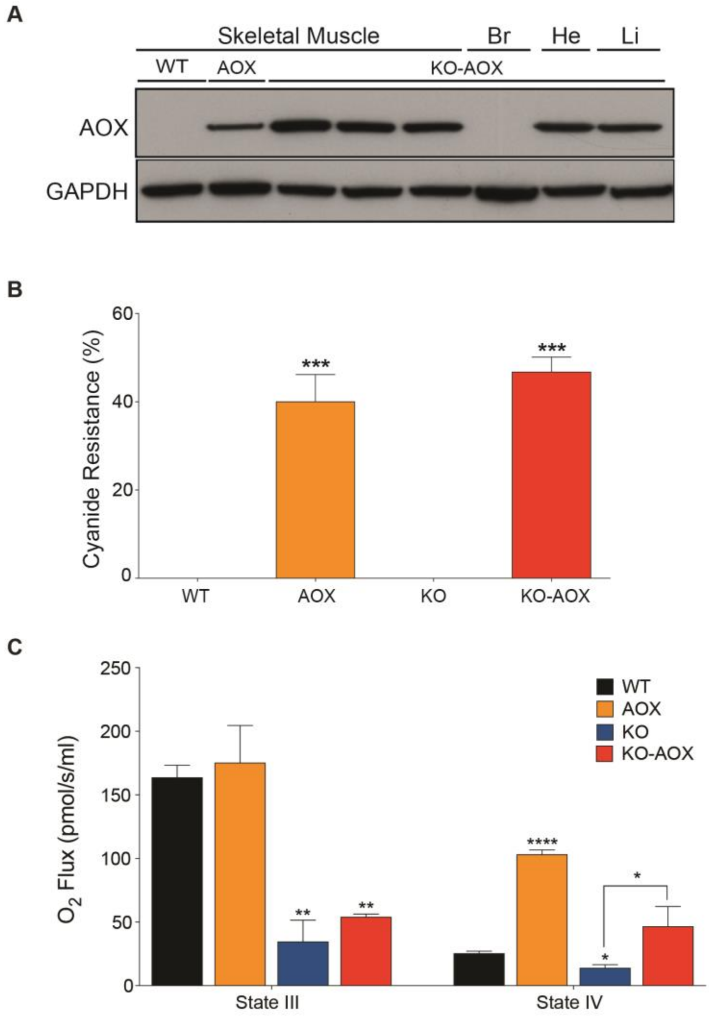

A
Skeletal Muscle
Br
He
Li
WT
AOX
KO-AOX
AOX
GAPDH
B
Cyanide Resistance (%)
60
40
20
0
***
***
WT
AOX
KO
KO-AOX
C
O2 Flux (pmol/s/ml)
250
200
150
100
50
0
WT
AOX
KO
KO-AOX
**
**
****
*
*
State III
State IV

Figure S4. Related to Figure 4. AOX is expressed and active in KO-AOX.

(A) Western blot analysis of AOX in skeletal muscle, brain (Br), heart (He) and liver (Li) of WT, AOX and KO-AOX mice. GAPDH is used as loading control. **(B)** Cyanide resistance in AOX and KO-AOX mice measured by percent of State III (ADP-stimulated) respiration (n=4). **(C)** Succinate-driven State III (ADP-stimulated) and State IV (oligomycin-sensitive) oxygen consumption rates in isolated skeletal muscle mitochondria (n = 4).

All experiments were done on 8-week-old animals. Bars represent mean ± S.E.M. Asterisks directly on top indicate level of statistical significance compared to WT (*p ≤ 0.05; **p ≤ 0.01; ***p ≤ 0.001; ****p < 0.0001; unpaired Student's *t*-test).

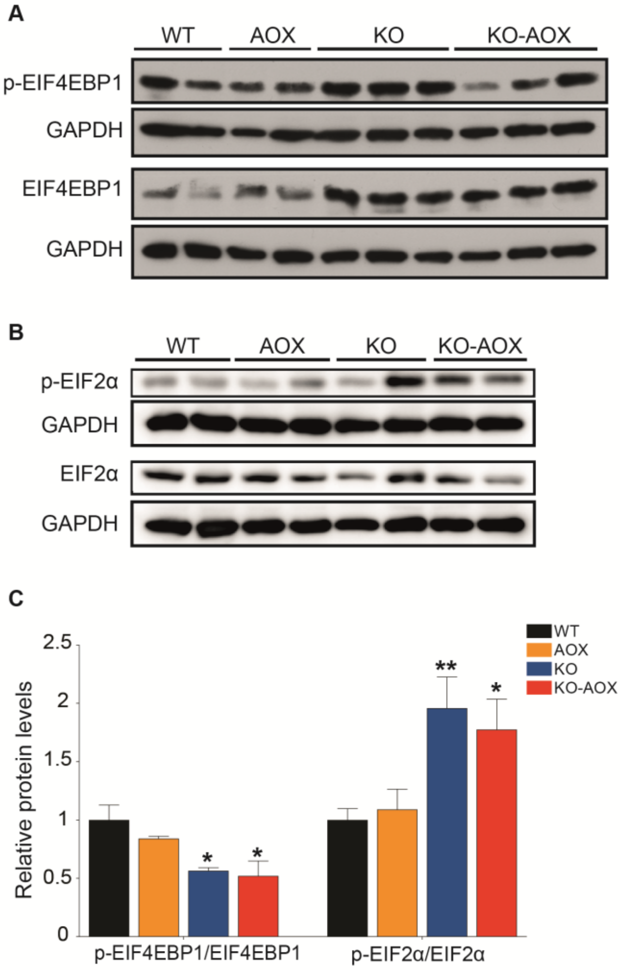
A
WT
AOX
KO
KO-AOX
p-EIF4EBP1
GAPDH
EIF4EBP1
GAPDH
B
WT
AOX
KO
KO-AOX
p-EIF2α
GAPDH
EIF2α
GAPDH
C
Relative protein levels
2.5
2
1.5
1
0.5
0
WT
AOX
KO
KO-AOX
*
*
**
*
p-EIF4EBP1/EIF4EBP1
p-EIF2α/EIF2α

Figure S5. Related to Figure 6. Both KO and KO-AOX show increased levels of EIF-2alpha (EIF2α) and decreased mTOR signaling.

Western blots of **(A)** phosphorylated- and total EIF4EBP1; and **(B)** phosphorylated- and total EIF2α. **(C)** Quantification (n = 5) of aforementioned proteins in WT, AOX, KO and KO-AOX mice.

Western blots were performed on skeletal muscle homogenates of 8-week-old mice. GAPDH is used as loading control. Bars represent mean ± S.E.M. Asterisks over the bars indicate statistical significance vs. WT; over the brackets among indicated groups. (*$p \leq 0.05$; **$p \leq 0.01$; unpaired Student's *t*-test).

Table S1. Related to Star Methods. qPCR primer sequences.

	Forward 5' - 3'	Reverse 5' - 3'
Afg3l2	GTTGATGGGCAATACGTCTGG	GACCCGGTTCTCCCCTTCT
Aldh18a1	AATCAGGGCCGAGAGATGATG	GGCCTCTAAGACCGGAATTGC
Atf3	CCAGAATAAACACCTCTGCCATCG	CTTCAGCTCAGCA TTCACACTCTC
Atf4	GCAAGGAGGATGCCTTTTC	GTTTCCAGGTCATCCATTCG
Atf5	CCTTGCCCTTGCCCACCTTTGAC	CCAGAGGAGGAGGCTGCTGT
Atf6	TCGCCTTTTAGTCCGGTTCTT	GGCTCCATAGGTCTGACTCC
Catalase	TGGCACACTTTGACAGAGAGC	CCTTTGCCTTGGAGTATCTGG
Chop	CTGGAAGCCTGGTATGAGGAT	CAGGGTCAAGAGTAGTGAAGGT
CoxI	TGCTAGCCGCAGGCATTACT	CGGGATCAAAGAAAGTTGTGTTT
CoxII	CAGGCCGACTAAATCAAGCAA	GAGCATTGGCCATAGAATAATCCT
CoxVa	TCATCCAGGAACTTAGACCAACT	AGTCCTTAGGAAGCCCATCG
Gpx1	CCACCGTGTATGCCTTCTCC	AGAGAGACGCGACATTCTCAAT
Hprt	TCCTCCTCAGACCGCTTTT	CCTGGTTCATCATCGCTAAT
mtHsp70	ATGGCTGGAATGGCCTTAGC	ACCCAAATCAATACCAACCACTG
Ndufs8	CTTCGGCTTTGTGGCTTTCATGGT	AAAGCCCATCAAGCCTCCTCAGAT
Nfe2l2	TCCATTCCCGAATTACAGTGTCT	GCCCACTTCTTTTTCCAGCG
Pycr1	ATGAGCGTAGGCTTCATCGG	GTGTCAGGTTCACCCCTATCT
RNaseP	GCCTACACTGGAGTCCGTGCTACT	CTGACCACACACGAGCTGGTAGAA
Sdhb	GACGTCAGGAGCCAAAATGG	CTCGACAGGCCTGAAACTGC
Sod1	CAAGCGGTGAACCAGTTGTG	TGAGGTCCTGCACTGGTAC
Sod2	GCCTGCACTGAAGTTCAATG	ATCTGTAAGCGACCTTGCTC
Uqcrfs1	ATCCCTGAAGGGAAGAACATGGCT	TGCAGCTTCCTGGTCAATCTCCTT
