# Supplementary material for: Perturbed Redox Signaling Exacerbates a Mitochondrial Myopathy
Source: Cell Metab. 2018 Nov 6;28(5):764–775.e5. doi: 10.1016/j.cmet.2018.07.012 (PMC6224544; doi:10.1016/j.cmet.2018.07.012)
Supplement: Document S2. Article plus Supplemental Information [file mmc2.pdf]

Article

Cell Metabolism

Perturbed Redox Signaling Exacerbates a Mitochondrial Myopathy

Graphical Abstract

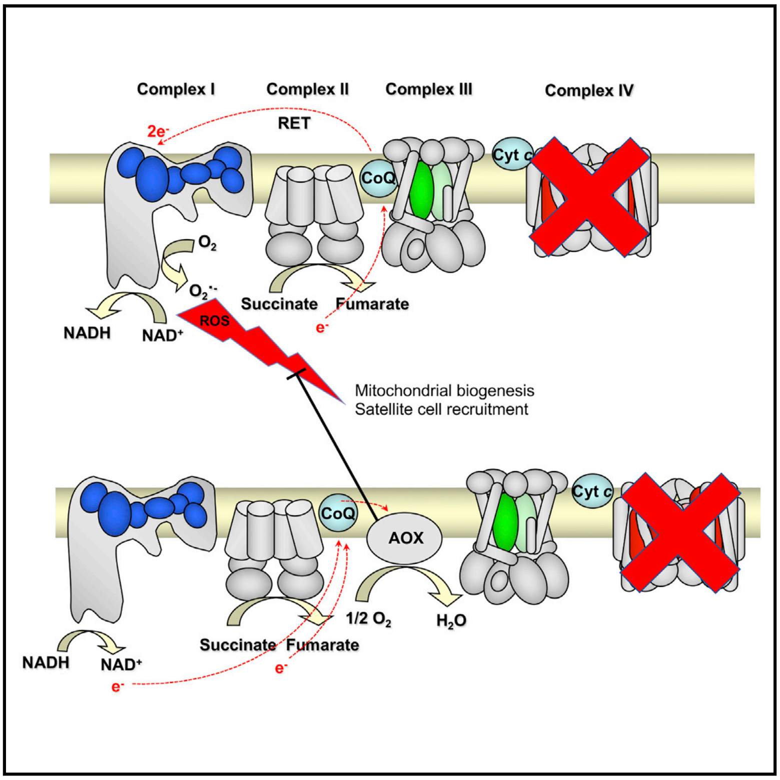

Highlights

- ROS trigger mitochondrial biogenesis in *Cox15*-deficient mice
- Satellite cell recruitment is ROS dependent in mitochondrial myopathy
- AOX and NAC interfere with ROS signaling
- The use of antioxidants in mitochondrial diseases should be carefully evaluated

Authors

Sukru Anil Dogan, Raffaele Cerutti, Cristiane Benincá, ..., Massimo Zeviani, Marten Szibor, Carlo Viscomi

Correspondence

marten.szibor@uta.fi (M.S.),
cfv23@mrc-mbu.cam.ac.uk (C.V.)

In Brief

Dogan et al. show that alternative oxidase attenuates ROS signaling in a COX-defective mitochondrial myopathy model, thus blunting ROS-dependent mitochondrial biogenesis and satellite cell recruitment. These findings must be considered in the treatment of mitochondrial myopathies, suggesting the need for careful assessment of antioxidant therapy.

Dogan et al., 2018, Cell Metabolism *28*, 764–775
November 6, 2018 © 2018 The Author(s). Published by Elsevier Inc.
https://doi.org/10.1016/j.cmet.2018.07.012

CellPress

Cell Metabolism
Article

Perturbed Redox Signaling Exacerbates a Mitochondrial Myopathy

Sukru Anil Dogan,[1] Raffaele Cerutti,[1] Cristiane Benincá,[1] Gloria Brea-Calvo,[2] Howard Trevor Jacobs,[3,4] Massimo Zeviani,[1] Marten Szibor,[3,4,*] and Carlo Viscomi[1,5,*]
[1]MRC Mitochondrial Biology Unit, University of Cambridge, Wellcome Trust/MRC Building Hills Road, Cambridge CB2 0XY, UK
[2]Centro Andaluz de Biología del Desarrollo and CIBERER, Instituto de Salud Carlos III, Universidad Pablo de Olavide-CSIC-JA, Sevilla 41013, Spain
[3]Institute of Biotechnology, University of Helsinki, Viikinkaari 5, Helsinki 00790, Finland
[4]Faculty of Medicine and Life Sciences, University of Tampere, Arvo Ylpön katu 34, Tampere 33520, Finland
[5]Lead Contact
*Correspondence: marten.szibor@uta.fi (M.S.), cfv23@mrc-mbu.cam.ac.uk (C.V.)
https://doi.org/10.1016/j.cmet.2018.07.012

SUMMARY

Alternative oxidases (AOXs) bypass respiratory complexes III and IV by transferring electrons from coenzyme Q directly to O_2. They have therefore been proposed as a potential therapeutic tool for mitochondrial diseases. We crossed the severely myopathic skeletal muscle-specific COX15 knockout (KO) mouse with an AOX-transgenic mouse. Surprisingly, the double KO-AOX mutants had decreased lifespan and a substantial worsening of the myopathy compared with KO alone. Decreased ROS production in KO-AOX versus KO mice led to impaired AMPK/PGC-1α signaling and PAX7/MYOD-dependent muscle regeneration, blunting compensatory responses. Importantly, the antioxidant *N*-acetylcysteine had a similar effect, decreasing the lifespan of KO mice. Our findings have major implications for understanding pathogenic mechanisms in mitochondrial diseases and for the design of therapies, highlighting the benefits of ROS signaling and the potential hazards of antioxidant treatment.

INTRODUCTION

Oxidative phosphorylation (OXPHOS) is the process by which mitochondria convert the energy derived from nutrients into ATP. Electrons generated by intermediary metabolism in the form of reducing equivalents are transferred along the four complexes of the mitochondrial respiratory chain (complexes I–IV, cI–cIV) to eventually combine with molecular oxygen to produce water. This exergonic process, termed respiration, sustains the extrusion of protons across the inner mitochondrial membrane, carried out by proton pumps present in cI, cIII, and cIV. Proton translocation generates an electrochemical gradient, giving rise to a membrane potential, $\Delta\psi$, which is exploited by the ATP synthase (complex V, cV) to convert ADP and Pi to ATP. Mutations in a vast array of genes encoded by either the nuclear or mitochondrial DNA (mtDNA) disrupt the respiratory chain and lead to primary mitochondrial diseases. Several interconnected mechanisms account for the cellular consequences of OXPHOS defects, including decreased ATP synthesis, increased production of reactive oxygen species (ROS), altered ion trafficking, deranged metabolite levels, and abnormalities in mitochondrial-related cell death and turnover pathways such as apoptosis and autophagy.

In particular, ROS are by-products of normal respiration, but can increase dramatically when the respiratory chain is impaired. ROS are in fact thought to play a "hormetic" double role: in physiological conditions, low levels of ROS act as signaling molecules regulating homeostatic pathways related to mitochondrial bioenergetics, whereas at high levels they act as toxic agents damaging cellular components, including nucleic acids, proteins, and lipids (Yun and Finkel, 2014). Along the respiratory chain, ROS are generated at different sites with cI, cII, and cIII playing the main role (Brand, 2016). In particular, cI generates ROS through reverse electron transfer (RET), which exploits the electrons flowing back from coenzyme Q (CoQ) when this is over-reduced by electrons from cII (Chouchani et al., 2014) or in the presence of drugs or genetic defects that inhibit cIII and/or cIV (Guarás et al., 2016). Although the detrimental role of ROS has recently been challenged (Scialò et al., 2016), cells have evolved highly efficient ROS scavenging systems, which in mammals are mainly controlled by an antioxidant response program, based on the Kelch-like ECH-associated protein 1 (KEAP1) and nuclear factor erythroid 2-related factor 2 (NRF2/NFE2L2) (Holmström and Finkel, 2014).

In spite of recent progress, no specific therapy is currently available for OXPHOS disorders. Because of their huge genetic heterogeneity, an effective therapy should have the widest possible applicability or at least have the potential to be applied to more than a single disease entity.

Alternative oxidases (AOXs) are cyanide-resistant, membrane-bound mitochondrial enzymes present in plants, lower eukaryotes, and some specific metazoan phyla, consisting of just a single gene product. AOXs maintain electron flow when the respiratory chain is inhibited at the level of cIII and/or cIV, by directly transferring electrons from CoQ to O_2, thus bypassing cIII and cIV and preventing over-reduction of the CoQ pool. Notably, AOX activity is not associated with proton pumping across the inner mitochondrial membrane and thus does not contribute

 © 2018 The Author(s). Published by Elsevier Inc.
This is an open access article under the CC BY license (http://creativecommons.org/licenses/by/4.0/).

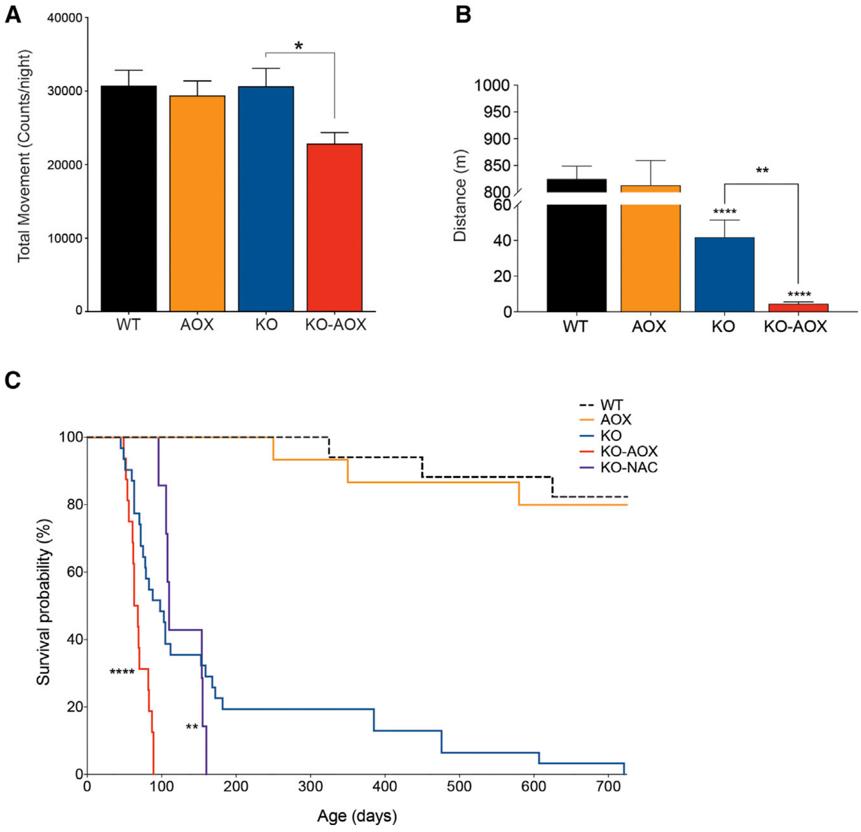

Figure 1. AOX Expression Exacerbates the Physical Properties and Lifespan of KO Mice

(A) Total movement of male 8-week-old WT, AOX, KO, and KO-AOX mice measured by CLAMS and indicated as counts per night (n = 8–10).

(B) Treadmill analysis of motor performance (n = 4).

(C) Kaplan-Meier survival curves (number of animals used are WT, 17; AOX, 15; KO, 31; KO-AOX, 16; KO-NAC, 8). Mean lifespans of KO-AOX and KO-NAC are compared with KO by one-sample t test. *N*-Acetylcysteine (NAC) was given to KO mice in the drinking water from 3 weeks of age.

Bars represent means ± SEM. Asterisks over the bars indicate statistical significance versus WT; over the brackets among indicated groups. *p ≤ 0.05; **p ≤ 0.01; ****p < 0.0001; unpaired Student's t test.

directly to the maintenance of $\Delta\psi$ and ATP synthesis. However, in the presence of cIII or cIV defects, the increase in proton pumping at cI, due to the re-activation of electron flow, should sustain the electrochemical gradient and ATP production. Importantly, the re-activation of electron flow by AOXs limits the excessive generation of ROS and maintains redox homeostasis, thereby maintaining tricarboxylic acid cycle activity (Mills et al., 2016). This has been exploited extensively to improve the phenotype of cellular and fly models with cIII and cIV defects (El-Khoury et al., 2014). However, its use in mammalian models has not been explored so far. Here, we report the *in vivo* effects of AOX expressed in a skeletal muscle-specific knockout mouse for *Cox15* (*Cox15*$^{sm/sm}$, hereafter designated KO), encoding the terminal enzyme of the biosynthetic pathway of *heme a*, an essential prosthetic group of cIV (cytochrome *c* oxidase [COX]).

RESULTS

AOX Expression Exacerbates the Phenotype of KO Mice

KO mice develop a profound, muscle-restricted COX deficiency leading to severe mitochondrial myopathy and early death (Viscomi et al., 2011). A mouse strain carrying *AOX* from the tunicate *Ciona intestinalis* inserted in the murine *Rosa26* locus has recently been described (AOX^{tg}, hereafter designated AOX), and was shown to be phenotypically indistinguishable from wild-type (WT) littermates (Szibor et al., 2016).

We crossed the KO and AOX lines to generate KO-AOX double mutants, to test whether AOX could alleviate the KO phenotype. Unexpectedly, KO-AOX mice showed a more severe phenotype, with earlier onset of symptoms than KO, characterized by decreased body weight (Figure S1A) due to diminished fat mass (Figure S1B) and decreased total spontaneous movements (Figure 1A) as well as treadmill motor performance in comparison with KO littermates (Figure 1B). The survival probability of the KO-AOX mice was markedly lower as well; its median lifespan was 60 days compared with 150 days for KO (log rank, $p < 0.0001$; Figure 1C). In fact, all the KO-AOX mice had to be euthanized by 90 days of age because of their poor condition.

Since the COX defect in KO mice is muscle specific, we reasoned that worsening of the myopathy might be responsible for the more drastic phenotype of KO-AOX mice. We thus analyzed the skeletal muscle from 8-week-old animals of the four genotypes, i.e., before KO-AOX mice start to die. COX/SDH histochemical staining confirmed the expected prevalence of COX-deficient fibers in KO versus WT and AOX animals. However, the defect was even more prominent in KO-AOX samples (Figure 2A). In addition, SDH staining was increased in KO muscles, but it was similar to the WT in KO-AOX mice (Figures S2A and S2B). Quantitative spectrophotometric assay of COX-specific activity in muscle homogenates confirmed this observation (10.29 ± 1.46 in KO versus 4.17 ± 0.47 in KO-AOX; $p < 0.01$; Figure 2B). Likewise, morphological analysis by hematoxylin and eosin (H&E) staining revealed significantly decreased cross-sectional area of KO-AOX versus KO myofibers (Figure 2C). The number of centralized nuclei, an index of skeletal muscle regeneration, was markedly increased in KO versus WT and AOX (8.55% ± 0.73% in KO versus 0.41% ± 0.13% in WT), but much less so in KO-AOX mice (Figure 2D). This finding prompted us to investigate the differentiation of satellite cells in the skeletal muscle of the different genotypes. The number of nuclei positive for PAX7, a marker of resident myoblasts, and MYOD, a marker of the differentiating satellite cells, was significantly increased in KO versus WT and AOX muscles, but it was expressed at normal levels in KO-AOX mice (Figures 3A and 3B).

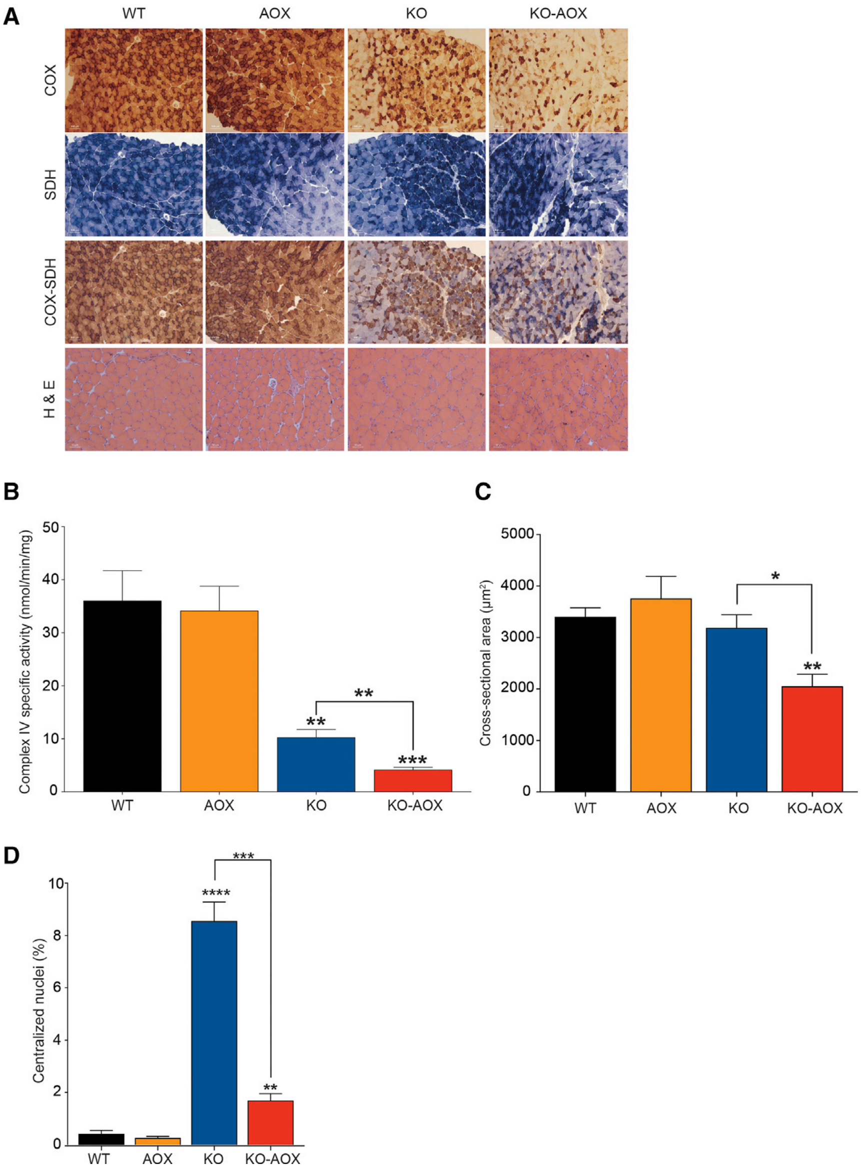

Figure 2. AOX Expression Worsens the Biochemical Muscle Phenotype of KO Mice

(A) Histochemical analyses of cytochrome *c* oxidase (COX), succinate dehydrogenase (SDH), double staining of COX-SDH, and H&E in 8-week-old WT, AOX, KO, and KO-AOX animals.

(B) Spectrophotometric specific activity of cIV in skeletal muscle of 8-week-old mice (n = 5).

(C) Analysis of the cross-sectional area of muscle fibers (n = 4).

(D) Analysis of the number of centralized nuclei in muscle fibers (n = 4).

Bars represent mean ± SEM. Asterisks over the bars indicate statistical significance versus WT; over the brackets among indicated groups. $^{*}p \leq 0.05$; $^{**}p \leq 0.01$; $^{***}p \leq 0.001$; $^{****}p < 0.0001$; unpaired Student's t test.

Next, we investigated if AOX determined a switch in the fiber type by immunodecorating muscle sections with antibodies against the different myosin isoforms. However, no differences were observed (Figures S3A and S3B). Taken together, these data clearly indicate that the mitochondrial myopathy was significantly more severe in KO-AOX animals.

Next, we confirmed AOX expression and catalytic activity in KO-AOX individuals. Western blot immunovisualization showed robust expression of AOX in most tissues except brain of adult mice, as previously reported (Szibor et al., 2016) (Figure S4A). Oxygraphic analysis of isolated mitochondria in the presence of ADP (state III) demonstrated substantial cyanide-resistant respiration in muscle of both AOX and double recombinant KO-AOX mice (Figure S4B). State III O_2 consumption rate was markedly higher in WT and AOX mitochondria compared with KO and KO-AOX. In contrast, oligomycin-sensitive respiration was markedly increased in both AOX and KO-AOX muscle mitochondria compared with the corresponding naive models, WT and KO, respectively (Figure S4C). These results indicate that AOX-dependent respiration is active but insensitive to either cyanide or oligomycin inhibition.

AOX Expression Interferes with Mitochondrial Biogenesis in KO-AOX

In addition to SDH staining, CS activity was also increased in KO animals compared with controls and KO-AOX (Figure 4A). These data prompted us to evaluate other markers of mitochondrial biogenesis in skeletal muscle. The mtDNA copy number (Figure 4B) and the expression levels of mitochondrial transcription factor A (TFAM) (Figure 4C) were increased in the KO versus WT and AOX but were similar to WT values in the double recombinant KO-AOX animals. In addition, the amount of several subunits of the respiratory complexes was significantly increased in KO versus WT and AOX, but not in samples from KO-AOX mice (Figure 4D). Overall, these data suggest that AOX expression blunts the increased mitochondrial biogenesis observed in the muscle-specific *Cox15*-defective model.

We then investigated whether the main mitochondriogenic control pathways were affected by AOX in KO muscles. AMP-dependent kinase (AMPK) is an important sensor of cellular energetic status and is activated when the AMP/ATP ratio increases (Lin and Hardie, 2017). Under such energy-deficient conditions, AMPK is activated by phosphorylation of Thr_{172} (p-AMPK) by LK1 kinase and, in turn, p-AMPK phosphorylates a large number of targets, including the transcriptional co-activator PGC-1α, a master regulator of mitochondrial biogenesis. As previously reported (Visconi et al., 2011), p-AMPK was upregulated in KO mice, but it was within the normal range in KO-AOX animals (Figure 4E). Moreover, PGC-1α protein amount was also increased in KO versus WT and AOX, while it was normal in KO-AOX muscle samples (Figure 4F). Finally, quantitative transcript

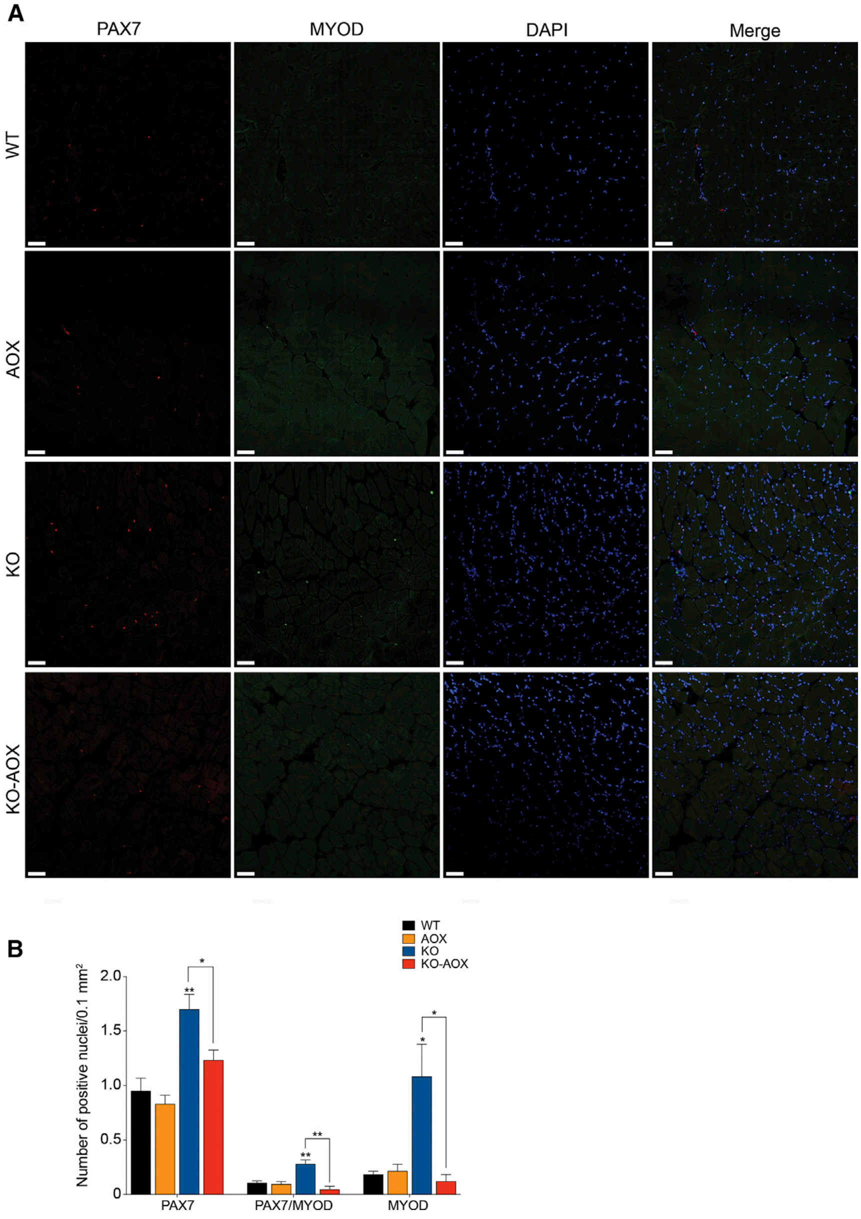

Figure 3. AOX Impairs the Regeneration Capacity of Myofibers

(A) Representative confocal 3D z stack image of 8-week-old muscle fibers labeled with PAX7 (red), MYOD (green), and DAPI (blue). The image represents a randomly chosen image from four samples. Scale bar, 50 μm.

(B) Quantification of the number of positive PAX7, PAX7/MYOD, and MYOD nuclei in muscles of WT, AOX, KO, and KO-AOX animals (n = 4). Bars represent means ± SEM. Asterisks over the bars indicate statistical significance versus WT; over the brackets among indicated groups. $^{*}p \leq 0.05$; $^{**}p \leq 0.01$; unpaired Student's t test.

analysis showed decreased expression of several genes related to the respiratory chain in KO-AOX versus KO mice, confirming that mitochondrial biogenesis is diminished in the presence of AOX (Figure 4G).

AOX Impairs ROS Signaling in KO Mice

Since AOX prevents excessive ROS production, we reasoned that it might interfere with ROS signaling in cIV-deficient muscle. Thus, we quantified ROS production in skeletal muscle mitochondria by measuring hydrogen peroxide (H_2O_2) production. Succinate-driven H_2O_2 production was significantly increased in KO compared with WT and AOX mitochondria but was lower in KO-AOX double mutants than in WT animals (Figure 5A). Accordingly, mitochondrial aconitase (ACO2) activity, which is inhibited by H_2O_2, was significantly lower in the KO compared with WT and AOX mitochondria but had normal values in the KO-AOX samples (Figure 5B). Altogether, these results are consistent with AOX being active only under stress conditions, as previously reported (Szibor et al., 2016).

Since AOX requires a highly reduced CoQ pool to be activated (Dry et al., 1989), we measured the amount of reduced and oxidized CoQ in frozen skeletal muscle of the mice. The relative amount of reduced CoQ was increased in KO versus WT and AOX mice but was comparable with WT in the double mutants, confirming that AOX efficiently oxidizes the CoQ pool (Figure 5C). These results support the idea that RET, which is promoted by over-reduction of the CoQ pool, is involved in generating ROS in the KO model, and that this phenomenon is blunted by AOX.

Increased ROS can trigger the oxidative stress response via KEAP1/NFE2L2 signaling. Accordingly, the transcripts for superoxide dismutase (*Sod2*) and glutathione peroxidase (*Gpx1*), two key enzymes of the antioxidant response that are NFE2L2 targets, were significantly increased in KO versus WT and AOX mice, but significantly decreased in KO-AOX versus KO animals (Figure 5D). A similar trend was detected for both *Cat*, encoding catalase, and *Sod1*, encoding the cytosolic isoform of superoxide dismutase (Figure 5D). A significant increase of the *Nfe2l2* transcript was also detected in KO but not in KO-AOX samples (Figure 5D).

Various retrograde signals from mitochondria can be activated under stress conditions associated with increased ROS, energy deficiency, and loss of $\Delta\psi$ (Quirós et al., 2016). As low levels of ATP can activate AMPK, as well as ROS (Rabinovitch et al., 2017), we assessed the ATP production rate. We found a strong but comparable impairment of ATP synthesis in both KO and KO-AOX skeletal muscle mitochondria, using either cI- or cII-linked substrates (Figure 5E). In addition, the ATP content was comparably lower than controls in KO and KO-AOX samples (Figure 5F). Likewise, $\Delta\psi$ was markedly but comparably lower than controls in both KO and KO-AOX muscle mitochondria

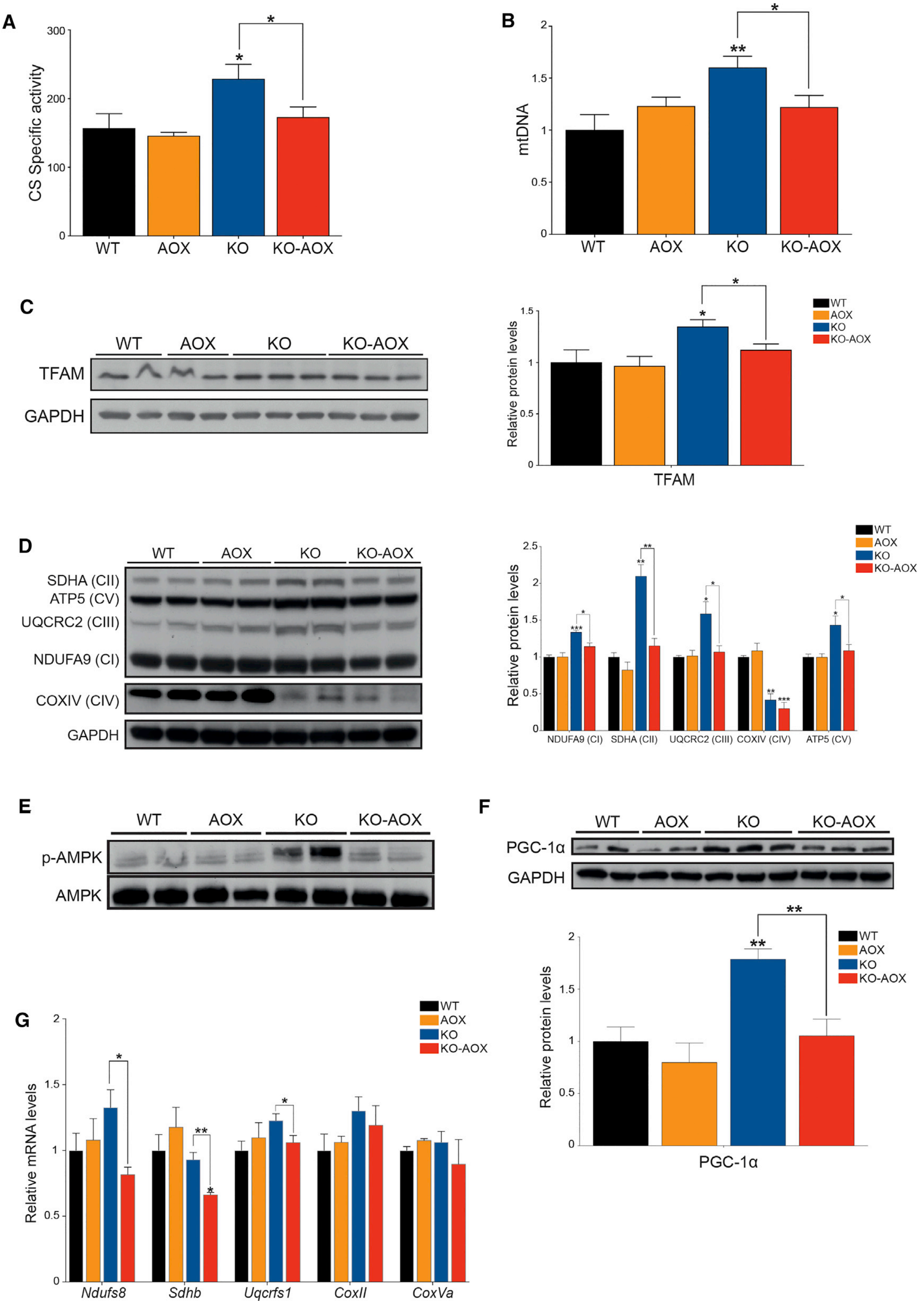

Figure 4. AOX Interferes with Mitochondrial Biogenesis in KO-AOX Mice

(A) Spectrophotometric activity of citrate synthase (CS)-specific activity (n = 5).

(B) mtDNA copy number by qPCR (n = 8–10).

(C) Western blot and quantification (n = 5) of TFAM.

(legend continued on next page)

(Figure 5G). The comparable decrease of ATP production rate, ATP steady-state levels, and $\Delta\psi$ in KO and KO-AOX indicates that impaired bioenergetics is not the main reason for the more severe phenotype of KO-AOX mice.

To further test the hypothesis that decreased ROS production underlies the aggravated phenotype, we supplemented the drinking water of eight KO mice after weaning with *N*-acetylcysteine (NAC), a cell-permeable precursor of glutathione (Viscomi et al., 2010). The mean survival was significantly shorter in treated versus untreated animals, and the maximal lifespan was grossly decreased in the NAC-treated cohort, although NAC supplementation also delayed the earliest deaths of KO mice (Figure 1A).

Autophagy Is Restored in KO-AOX Skeletal Muscle

Mitochondrial biogenesis and autophagy together regulate mitochondrial content (Peralta et al., 2016). We measured the amount of LC3, a marker for autophagosomes, and P62, a marker for autophagic cargoes, in skeletal muscle samples of the different models. The ratio between lipidated, autophagosome-associated LC3 (LC3-II) and non-lipidated, cytosolic free LC3 (LC3-I) was significantly decreased, whereas the amount of P62 was increased, in KO versus WT and AOX muscles, suggesting decreased autophagy (Figure 6A). Conversely, KO-AOX muscle showed a markedly increased LC3-II/LC3-I ratio while the levels of P62 were comparable with WT, indicating no decrease in autophagy.

Mitochondrial Stress Markers Are Comparably Increased in Both KO and KO-AOX

A number of mitochondrial pathways are activated in response to mitochondrial dysfunction. We measured the expression at the protein and/or mRNA level, of several components of this integrated stress response, including (1) key players of the mitochondrial unfolded protein response (UPR^{mt}) (Shpilka and Haynes, 2017), such as the bZIP transcription factors *Chop* (C/EBP-homologous protein), *Atf4*, and *Atf5* (Fiorese et al., 2016), and mitochondrial chaperones HSP60/HSPD1 and mtHSP70/HSPA9 (Dogan et al., 2014); (2) components of the one-carbon/mitochondrial folate cycle, e.g., methylene tetrahydrofolate dehydrogenase 2 (MTHFD2) (Khan et al., 2017; Kuhl et al., 2016), and enzymes of proline biosynthesis from glutamate, e.g., delta-1-pyrroline-5-carboxylate synthase (ALDH18A1) and mitochondrial pyrroline-5-carboxylate reductase 1 (PYCR1); and (3) the fibroblast growth factor 21 (*Fgf21*) and growth differentiation factor 15 (*Gdf15*) mitokines (Lehtonen et al., 2016). Increased amounts of these markers or their transcripts were detected in both KO and KO-AOX animals (Figures 6B–6D). In particular, *Fgf21* mRNA was highly expressed in KO muscles, and even more so (260-fold) in KO-AOX mice (Figure 6D). *Gdf15* was about 100-fold higher than controls in KO mice, but significantly lower in KO-AOX, although still increased relative to WT animals (Figure 6D). Other factors involved in the integrated stress response, including *Atf3* and *Atf6*, related respectively to cell death and ER-stress pathways, did not change (*Atf3*) or changed to a very small extent (*Atf6*). Interestingly, the activation of these stress-related pathways in our KO model takes place via EIF-2alpha and not via mTORC1 (Melber and Haynes, 2018). The levels of mTORC1 target eukaryotic translation initiation factor-binding protein 1 (EIF4EBP1), an important player in protein synthesis (Morita et al., 2013) was increased in both KO and KO-AOX compared with WT. However, phosphorylated versus unphosphorylated EIF4EBP1 ratio was decreased to the same extent in KO and KO-AOX versus WT and AOX mice, indicating that mTORC1 signaling, and thus protein synthesis and cell growth, was reduced in both KO and KO-AOX muscles. In contrast, we detected increased levels of phosphorylated EIF-2alpha in both KO and KO-AOX mice compared with both WT and AOX littermates (Figures S5A–S5C). These findings suggest that the induction of major mitochondrial stress responses correlates with mitochondrial myopathy and disease progression but AOX did not significantly modify these pathways, except insofar as it worsened the myopathy, affecting the expression of relevant endocrine markers, such as *Gdf15* (Figure 6E).

DISCUSSION

While the expression of an AOX xenogene on a murine WT background had hardly any consequences, we here showed that AOX entrains a dramatic worsening of the clinical and biochemical phenotype in a mouse model of COX-defective mitochondrial myopathy. Under stress conditions, including defects of cIII or cIV, AOX directly oxidizes CoQ, maintaining electron flow from NADH and $FADH_2$ but abolishing the contribution of cIII and cIV to the formation of the $\Delta\psi$. The unexpected outcome observed in the KO-AOX double mutant model suggested two possible mechanisms: a direct bioenergetic failure caused by the exclusion of the proton pumping activity of cIII and cIV, or an indirect effect consequent to decreased ROS signaling and blunting of mitochondrial biogenesis. We excluded the first possibility, since no difference was detected in the ATP production rate, ATP levels, or $\Delta\psi$ between KO and KO-AOX muscle samples, as expected since AOX does not translocate protons across the mitochondrial inner membrane. Conversely, ROS production was significantly decreased in KO-AOX muscle as were several indicators of mitochondrial biogenesis.

Redox signaling controls a large number of transcriptional pathways (Holmström and Finkel, 2014). However, its importance *in vivo* has not been adequately investigated. While our results argue against the general applicability of AOX as a therapeutic tool in mitochondrial disorders, they provide solid genetic

(D) Western blot and quantification (n = 5) for MRC complexes. Individual subunits and the complexes are indicated on the left.

(E) Representative western blot for phosphorylated and total AMPK.

(F) Western blot and quantification (n = 5) of PGC-1α.

(G) Relative expression levels of respiratory chain transcripts (n = 6). Results represent fold increase normalized against WT.

All experiments were performed on 8-week-old mice with western blots using skeletal muscle homogenates of 8-week-old mice and GAPDH as loading control. Bars represent means ± SEM. Asterisks over the bars indicate statistical significance versus WT; over the brackets among indicated groups. *$p \leq 0.05$; **$p \leq 0.01$; ***$p \leq 0.001$; unpaired Student's t test.

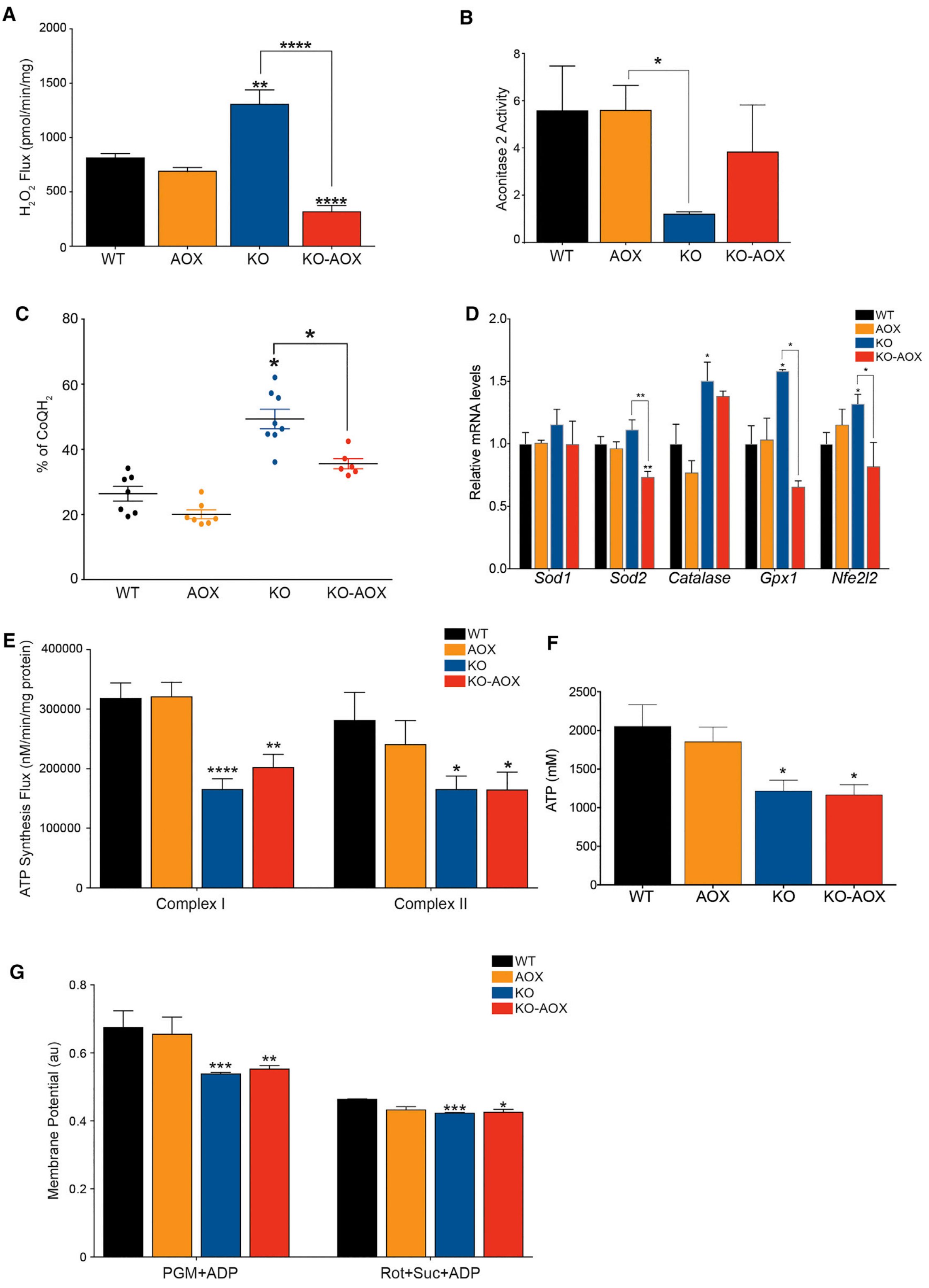

Figure 5. AOX Impairs ROS Signaling in KO Mice

(A) H_2O_2 production rate caused by RET in isolated skeletal muscle mitochondria (n = 4).

(B) Aconitase2 activity in frozen skeletal muscle samples (n = 5).

(C) Percentage of reduced CoQ in frozen muscle samples.

(D) Relative expression levels of antioxidant response transcripts (n = 6). Results represent fold increase normalized against WT.

(legend continued on next page)

evidence in a mammalian mitochondrial disease model of the crucial pathophysiological role of ROS-dependent pathways as compensatory responses to mitochondrial dysfunction, at least in skeletal muscle, as previously suggested (Horn et al., 2017; Reczek and Chandel, 2015). This has obvious implications in understanding some of the pathogenic mechanisms of mitochondrial diseases and can potentially be exploited to develop effective therapies. For instance, an ROS-based mechanism can underpin the formation of ragged-red fibers (RRFs), a morphological hallmark of mitochondrial myopathy. RRFs are determined by the segmental accumulation of dysfunctional mitochondria along the muscle syncytium, especially in the sub-sarcolemmal region. However, the mechanism leading to their formation is poorly understood. We propose that ROS can be a major signal inducing the local proliferation of mitochondria, in an attempt to compensate their functional defect through the activation of a mitochondriogenic program by the surrounding nuclei. Furthermore, our findings support the idea that the induction of mitochondrial biogenesis, for instance by activating the SIRT1-and/or AMPK-dependent PGC-1α axis, or other mitochondriogenic pathways, could be a rational and potentially effective approach in the therapy of mitochondrial diseases. Interestingly, there is increasing evidence that AMPK is directly regulated by ROS either through oxidation and S-glutathionylation of cysteines 299 and 304 in the α subunit, leading to the activation of the enzyme, or oxidation of cysteines 130 and 174 in the α subunit, resulting in its inactivation (Shao et al., 2014). An additional mechanism potentially contributing to the decrease of mitochondrial content is the maintenance of autophagic flux in KO-AOX, as suggested by an increased LC3-II/LC3-I ratio and normalization of the P62 level, which is impaired in KO muscle. Further investigation will be needed to understand whether this effect is directly related to decreased ROS production or is an indirect consequence of the activation of stress responses. The integrated stress response is indeed activated in both KO and KO-AOX mice. This seems to be controlled by the activation of the translation initiation factor EIF-2alpha by phosphorylation operated by GCN2 or other kinases under mitochondrial stress conditions (Melber and Haynes, 2018) and as observed in other mouse models of mitochondrial dysfunction (Seiferling et al., 2016). In contrast, mTORC1 signaling, which was shown to regulate mitochondrial integrated stress response in a model of impaired mtDNA replication (Khan et al., 2017), is inhibited in both KO and KO-AOX mice as indicated by reduced levels of phosphorylated EIF4EBP1. Interestingly, we found that two mitokines recently introduced as biomarkers for mitochondrial myopathies (Lehtonen et al., 2016) were both highly increased in KO and KO-AOX mice. However, while *Fgf21* was much higher in KO-AOX than in KO, *Gdf15* was reduced in KO-AOX compared with KO, implying a stress-dependent modulation of the latter, as suggested by previous work (Chung et al., 2017).

An intriguing finding of our study is the decrease in the number of centralized nuclei in KO-AOX versus KO muscle fibers. Centralized nuclei are a primary sign of muscle regeneration (Yin et al., 2013). During muscle regeneration, satellite cells, which constitute the resident pool of stem cells in skeletal muscle, undergo a process of activation, characterized by proliferation and migration to the site of damage, where they differentiate into myocytes and eventually fuse with the existing myofibers. Specific transcription factors are expressed during this process, including *Pax7*, *MyoD*, and *Myogenin*. Our data suggest that ROS influence this pathway, similar to what is observed in other populations of stem cells. Myogenic differentiation is associated with high ROS levels, mainly due to the concomitant induction of mitochondrial biogenesis (L'honoré et al., 2014). Antioxidant defenses are activated at the same time to prevent cellular damage (Latella and Puri, 2014). The decreased number of centralized nuclei and reduced PAX7- and MYOD-positive nuclei in KO-AOX muscles indicate that satellite cells are present but cannot differentiate into myotubes, thus impairing the capacity for repair/regeneration of the myofibers. This effect is likely to have an important role in the observed worsening of the phenotype in KO-AOX mice.

The majority of ongoing clinical trials for mitochondrial diseases are based on the use of antioxidants, stemming from the assumption that excessive ROS production causes oxidative damage to cellular components. However, there has hitherto been only a minimal investigation of the extent of ROS production and oxidative damage in mitochondrial diseases *in vivo*, and the available data are contradictory. For instance, in the mutator mouse (Trifunovic et al., 2004), no signs of oxidative damage have been detected in post-mitotic tissues (Trifunovic et al., 2005), while extensive ROS damage has been shown in replicating cells (Hamalainen et al., 2015). Our data using both AOX and NAC highlight a potential risk associated with antioxidant use, of interfering with compensatory ROS signaling, at least in mitochondrial myopathies. This observation is in agreement with previous reports showing that antioxidants can have deleterious effects on pluripotent stem cells with impaired mitochondrial function (Hamalainen et al., 2015). However, it should be noted that NAC and AOX act on redox homeostasis through different mechanisms, as AOX prevents excess ROS production by increasing electron flow along the respiratory chain and is expressed transgenically throughout embryonic development, while NAC, a glutathione precursor, was administered shortly after weaning, thus explaining the observed differences in their survival curves.

In our model, COX deficiency leads to accumulation of the reduced form of CoQ, and thus to excess production of superoxide anion via RET. By re-activating the electron flow along the respiratory chain, AOX normalizes the CoQ pool and abolishes the increases in RET and ROS production. Our data indicate that this effect, in turn, precludes the activation of a number of

(E) ATP synthesis flux in skeletal muscle mitochondria in the presence of cI-linked substrates (pyruvate, malate, glutamate) + ADP or cII-linked substrate (succinate, rotenone) + ADP (n = 4).

(F) ATP content in frozen skeletal muscle samples (n = 6–8).

(G) Analysis of mitochondrial membrane potential using safranin in isolated skeletal muscle mitochondria (n = 4).

All experiments were performed on 8-week-old mice. Bars represent means ± SEM. Asterisks over the bars indicate statistical significance versus WT; over the brackets among indicated groups. *$p \leq 0.05$; **$p \leq 0.01$; ***$p \leq 0.001$; ****$p < 0.0001$; unpaired Student's t test.

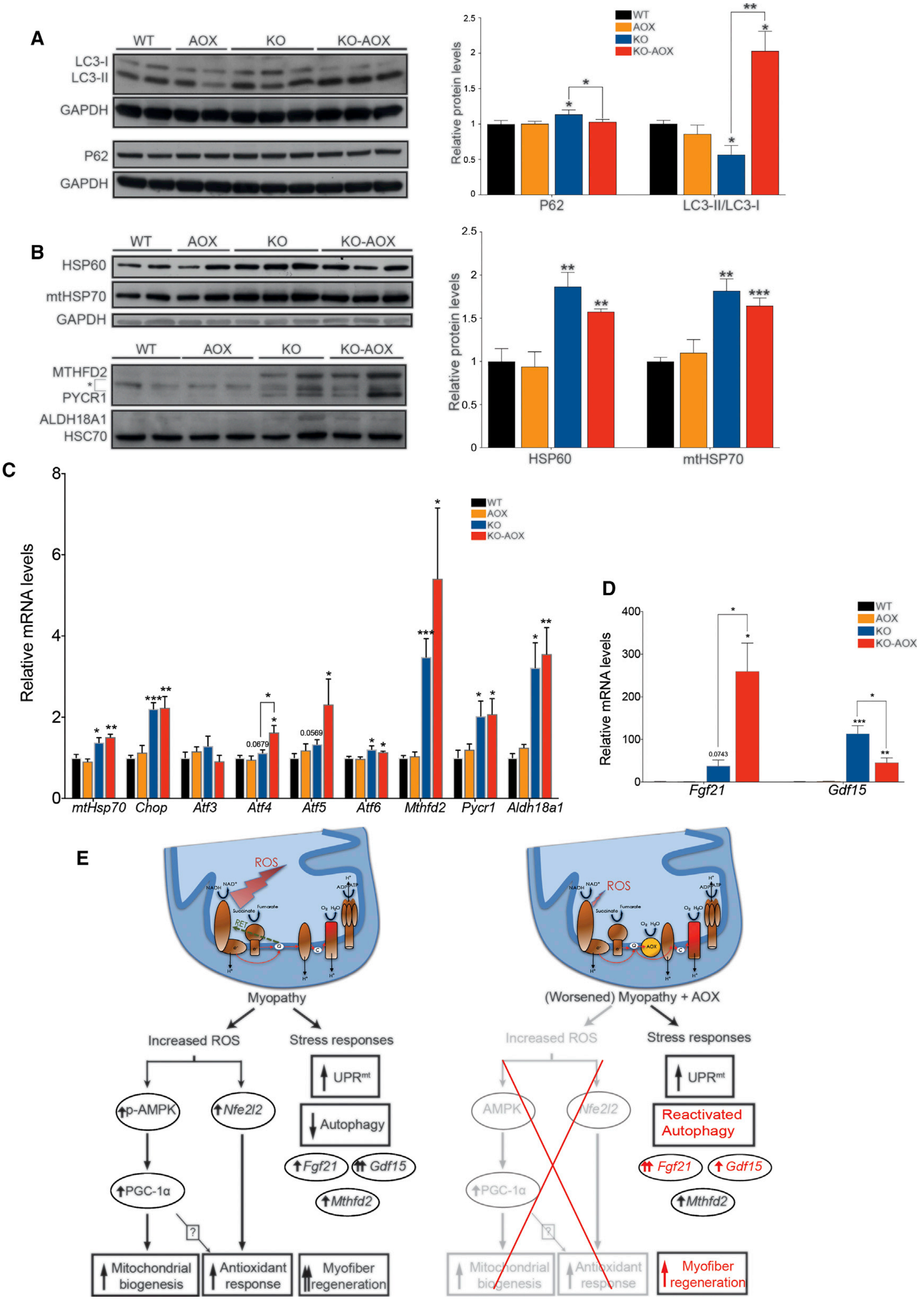

Figure 6. Mitochondrial Stress Responses Are Upregulated Regardless of AOX Expression, Except for Autophagy

(A) Western blots and quantification (n = 10) of autophagy markers (P62, LC3-I, and -II).

(B) Western blots and quantification (n = 10) of proteins involved in UPR^{mt} (HSP60 and mtHSP70) and mitochondrial metabolism (MTHFD2, PYCR1, and ALDH18A1). *Unspecific protein.

(legend continued on next page)

transcriptional response pathways and networks, as confirmed by real-time PCR data. Thus, AOX (or antioxidants) can block the homeostatic response to bioenergetic failure, exacerbating the pathological phenotype.

Our data do not rule out the possibility that AOX may be beneficial in other conditions affecting different organs and/or due to different mutations, which impair the activities of cIII and/or cIV or affect multiple respiratory complexes. It is also worth noting that not all mitochondrial diseases are associated with increased mitochondrial biogenesis. In addition, ROS produced at different sites may have different impact on whether their signaling or toxic role prevails (Sanz, 2016). In particular, it has recently been shown that ROS produced selectively via RET increases lifespan in *Drosophila*, whereas an opposite effect is observed when RET is prevented by increasing CoQ oxidation via AOX expression (Scialò et al., 2016), suggesting that over-reduction of CoQ generates an ROS signal important for homeostasis via RET. Along the same lines, we showed here that the accumulation of over-reduced CoQ increases ROS production via RET, triggering complex transcriptional cascades, which control mitochondria-related pathways to alleviate the pathology. Our findings thus have important consequences for the future treatment of mitochondrial disorders.

Limitations of the Study

Our work is limited to a single antioxidant compound in a single COX-defective myopathy. Antioxidants are widely used in the therapy of mitochondrial diseases and, although there is weak evidence of their efficacy, toxicity is usually not a major concern. We cannot exclude that in conditions associated with cIII or cIV deficiency in which ROS overproduction plays a major pathogenic role, the introduction of AOX may be beneficial.

STAR★METHODS

Detailed methods are provided in the online version of this paper and include the following:

- KEY RESOURCES TABLE
- CONTACT FOR REAGENT AND RESOURCE SHARING
- EXPERIMENTAL MODEL AND SUBJECT DETAILS
- METHOD DETAILS
  - Histochemistry, Immunofluorescence and Imaging
  - Analysis of Mitochondrial Enzyme Activities
  - Analysis of Body Composition
  - Real-Time Quantitative Polymerase Chain Reaction
  - Western Blot Analysis
  - Mitochondria Isolation and H_2O_2 Production
  - Mitochondrial Aconitase Activity
  - QH_2/Q Measurements
  - Measurement of ATP Synthesis Flux and ATP Content
  - Mitochondrial Membrane Potential Measurement
- QUANTIFICATION AND STATISTICAL ANALYSIS

SUPPLEMENTAL INFORMATION

Supplemental Information includes five figures and one table and can be found with this article online at https://doi.org/10.1016/j.cmet.2018.07.012.

ACKNOWLEDGMENTS

The authors wish to thank Ana Belen Cortes for technical assistance and Prof. Anthony Moore, University of Sussex, for helpful discussion. This work was supported by MRC-QQR Grant 2015-2020 (M.Z.), ERC Advanced Grants ERC FP7-322424 (M.Z.) and 232738 (H.T.J.), NRJ-Institut de France (M.Z.), and Academy of Finland grants 272376 and 256615 (H.T.J.). S.A.D. was supported by an EMBO Long-Term Fellowship (ALTF 856-2014) and EU (LTFCOFUND2013, GA-2013-609409). We are grateful to the personnel at ARES and Phenomics Animal Care Facilities for the support in managing our colonies. The BF-F3, SC-71, and BA-F8 monoclonal antibodies developed by Prof. Stefano Schiaffino, University of Padova, Italy, were obtained from the Developmental Studies Hybridoma Bank; created by the NICHD of the NIH; and maintained at The University of Iowa, Department of Biology, Iowa City, IA 52242.

AUTHOR CONTRIBUTIONS

S.A.D. performed all the experiments except histochemical and morphometric analyses (by R.C. and C.B.) and QH_2/Q measurements (by G.B.-C.). The study was conceived, planned, and supervised by C.V., H.T.J., M.S., and M.Z. All authors contributed to writing and commenting on the manuscript.

DECLARATION OF INTERESTS

The authors declare no competing interests.

Received: February 9, 2018
Revised: May 18, 2018
Accepted: July 18, 2018
Published: August 16, 2018

REFERENCES

Brand, M. (2016). Mitochondrial generation of superoxide and hydrogen peroxide as the source of mitochondrial redox signaling. Free Radic. Biol. Med. *100*, 14–31.

Bugiani, M., Invernizzi, F., Alberio, S., Briem, E., Lamantea, E., Carrara, F., Moroni, I., Farina, L., Spada, M., Donati, M.A., et al. (2004). Clinical and molecular findings in children with complex I deficiency. Biochim. Biophys. Acta *1659*, 136–147.

Chouchani, E., Pell, V., Gaude, E., Aksentijević, D., Sundier, S., Robb, E., Logan, A., Nadtochiy, S., Ord, E., Smith, A., et al. (2014). Ischaemic accumulation of succinate controls reperfusion injury through mitochondrial ROS. Nature *515*, 431–435.

Chowdhury, S., Djordjevic, J., Albensi, B., and Fernyhough, P. (2015). Simultaneous evaluation of substrate-dependent oxygen consumption rates

(C and D) Relative expression levels of mitochondrial stress response transcripts (n = 6). Results represent fold increase compared with WT that was normalized to 1.

(E) Proposed model for activation of mitochondrial biogenesis and different stress responses in *Cox15*$^{sm/sm}$ skeletal muscle. AOX expression worsens the myopathy. Some stress responses, emphasized in red, show differences compared with KO mice. The arrows correspond to the levels of induction of the specified markers (one arrow, moderate increase; two arrows, considerable increase).

Western blot were performed on skeletal muscle homogenates of 8-week-old mice. GAPDH and HSC70 were used as loading controls. Bars represent means ± SEM. Asterisks/p values over the bars indicate statistical significance versus WT; over the brackets among indicated groups. *$p \leq 0.05$; **$p \leq 0.01$; ***$p \leq 0.001$; unpaired Student's t test.

and mitochondrial membrane potential by TMRM and safranin in cortical mitochondria. Biosci. Rep. *36*, e00286.

Chung, H.K., Ryu, D., Kim, K.S., Chang, J.Y., Kim, Y.K., Yi, H.S., Kang, S.G., Choi, M.J., Lee, S.E., Jung, S.B., et al. (2017). Growth differentiation factor 15 is a myomitokine governing systemic energy homeostasis. J. Cell Biol. *216*, 149–165.

Dogan, S., Pujol, C., Maiti, P., Kukat, A., Wang, S., Hermans, S., Senft, K., Wibom, R., Rugarli, E., and Trifunovic, A. (2014). Tissue-specific loss of DARS2 activates stress responses independently of respiratory chain deficiency in the heart. Cell Metab. *19*, 458–469.

Dry, I.B., Moore, A.L., Day, D.A., and Wiskich, J.T. (1989). Regulation of alternative pathway activity in plant mitochondria: nonlinear relationship between electron flux and the redox poise of the quinone pool. Arch. Biochem. Biophys. *273*, 148–157.

El-Khoury, R., Kemppainen, K., Dufour, E., Szibor, M., Jacobs, H., and Rustin, P. (2014). Engineering the alternative oxidase gene to better understand and counteract mitochondrial defects: state of the art and perspectives. Br. J. Pharmacol. *171*, 2243–2249.

Fernandez-Marcos, P., Jeninga, E., Canto, C., Harach, T., de Boer, V., Andreux, P., Moullan, N., Pirinen, E., Yamamoto, H., Houten, S., et al. (2012). Muscle or liver-specific Sirt3 deficiency induces hyperacetylation of mitochondrial proteins without affecting global metabolic homeostasis. Sci. Rep. *2*, 425.

Fiorese, C., Schulz, A., Lin, Y., Rosin, N., Pellegrino, M., and Haynes, C. (2016). The transcription factor ATF5 mediates a mammalian mitochondrial UPR. Curr. Biol. *26*, 2037–2043.

Frezza, C., Cipolat, S., and Scorrano, L. (2007). Organelle isolation: functional mitochondria from mouse liver, muscle and cultured fibroblasts. Nat. Protoc. *2*, 287–295.

Guarás, A., Perales-Clemente, E., Calvo, E., Acín-Pérez, R., Loureiro-Lopez, M., Pujol, C., Martínez-Carrascoso, I., Nuñez, E., García-Marqués, F., Rodríguez-Hernández, M., et al. (2016). The CoQH2/CoQ ratio serves as a sensor of respiratory chain efficiency. Cell Rep. *15*, 197–209.

Hamalainen, R.H., Ahlqvist, K.J., Ellonen, P., Lepisto, M., Logan, A., Otonkoski, T., Murphy, M.P., and Suomalainen, A. (2015). mtDNA mutagenesis disrupts pluripotent stem cell function by altering redox signaling. Cell Rep. *11*, 1614–1624.

Holmström, K., and Finkel, T. (2014). Cellular mechanisms and physiological consequences of redox-dependent signalling. Nat. Rev. Mol. Cell Biol. *15*, 411–421.

Horn, A., Van der Meulen, J.H., Defour, A., Hogarth, M., Sreetama, S.C., Reed, A., Scheffer, L., Chandel, N.S., and Jaiswal, J.K. (2017). Mitochondrial redox signaling enables repair of injured skeletal muscle cells. Sci. Signal. *10*, https://doi.org/10.1126/scisignal.aaj1978.

Khan, N.A., Nikkanen, J., Yatsuga, S., Jackson, C., Wang, L., Pradhan, S., Kivela, R., Pessia, A., Velagapudi, V., and Suomalainen, A. (2017). mTORC1 regulates mitochondrial integrated stress response and mitochondrial myopathy progression. Cell Metab. *26*, 419–428.e5.

Krumschnabel, G., Fontana-Ayoub, M., Sumbalova, Z., Heidler, J., Gauper, K., Fasching, M., and Gnaiger, E. (2015). Simultaneous high-resolution measurement of mitochondrial respiration and hydrogen peroxide production. Methods Mol. Biol. *1264*, 245–261.

Kuhl, I., Miranda, M., Posse, V., Milenkovic, D., Mourier, A., Siira, S.J., Bonekamp, N.A., Neumann, U., Filipovska, A., Polosa, P.L., et al. (2016). POLRMT regulates the switch between replication primer formation and gene expression of mammalian mtDNA. Sci. Adv. *2*, e1600963.

L'honoré, A., Commère, P., Ouimette, J., Montarras, D., Drouin, J., and Buckingham, M. (2014). Redox regulation by Pitx2 and Pitx3 is critical for fetal myogenesis. Dev. Cell *29*, 392–405.

Latella, L., and Puri, P. (2014). Redox or death: checking on fetal myogenesis. Dev. Cell *29*, 373–374.

Lehtonen, J.M., Forsstrom, S., Bottani, E., Viscomi, C., Baris, O.R., Isoniemi, H., Hockerstedt, K., Osterlund, P., Hurme, M., Jylhava, J., et al. (2016). FGF21 is a biomarker for mitochondrial translation and mtDNA maintenance disorders. Neurology *87*, 2290–2299.

Lin, S., and Hardie, D. (2017). AMPK: sensing glucose as well as cellular energy status. Cell Metab. *27*, 299–313.

Melber, A., and Haynes, C.M. (2018). UPR(mt) regulation and output: a stress response mediated by mitochondrial-nuclear communication. Cell Res. *28*, 281–295.

Mills, E.L., Kelly, B., Logan, A., Costa, A.S.H., Varma, M., Bryant, C.E., Tourlomousis, P., Dabritz, J.H.M., Gottlieb, E., Latorre, I., et al. (2016). Succinate dehydrogenase supports metabolic repurposing of mitochondria to drive inflammatory macrophages. Cell *167*, 457–470.e13.

Morita, M., Gravel, S.P., Chenard, V., Sikstrom, K., Zheng, L., Alain, T., Gandin, V., Avizonis, D., Arguello, M., Zakaria, C., et al. (2013). mTORC1 controls mitochondrial activity and biogenesis through 4E-BP-dependent translational regulation. Cell Metab. *18*, 698–711.

Mourier, A., Motori, E., Brandt, T., Lagouge, M., Atanassov, I., Galinier, A., Rappl, G., Brodesser, S., Hultenby, K., Dieterich, C., et al. (2015). Mitofusin 2 is required to maintain mitochondrial coenzyme Q levels. J. Cell Biol. *208*, 429–442.

Peralta, S., Garcia, S., Yin, H., Arguello, T., Diaz, F., and Moraes, C. (2016). Sustained AMPK activation improves muscle function in a mitochondrial myopathy mouse model by promoting muscle fiber regeneration. Hum. Mol. Genet. *25*, 3178–3191.

Quirós, P., Mottis, A., and Auwerx, J. (2016). Mitonuclear communication in homeostasis and stress. Nat. Rev. Mol. Cell Biol. *17*, 213–226.

Rabinovitch, R., Samborska, B., Faubert, B., Ma, E., Gravel, S., Andrzejewski, S., Raissi, T., Pause, A., St-Pierre, J., and Jones, R. (2017). AMPK maintains cellular metabolic homeostasis through regulation of mitochondrial reactive oxygen species. Cell Rep. *21*, 1–9.

Reczek, C.R., and Chandel, N.S. (2015). ROS-dependent signal transduction. Curr. Opin. Cell Biol. *33*, 8–13.

Rodríguez-Aguilera, J., Cortés, A., Fernández-Ayala, D., and Navas, P. (2017). Biochemical assessment of coenzyme Q10 deficiency. J. Clin. Med. *6*, https://doi.org/10.3390/jcm6030027.

Sanz, A. (2016). Mitochondrial reactive oxygen species: do they extend or shorten animal lifespan. Biochim. Biophys. Acta *1857*, 1116–1126.

Schindelin, J., Arganda-Carreras, I., Frise, E., Kaynig, V., Longair, M., Pietzsch, T., Preibisch, S., Rueden, C., Saalfeld, S., Schmid, B., et al. (2012). Fiji: an open-source platform for biological-image analysis. Nat. Methods *9*, 676–682.

Scialò, F., Sriram, A., Fernández-Ayala, D., Gubina, N., Lõhmus, M., Nelson, G., Logan, A., Cooper, H., Navas, P., Enríquez, J., et al. (2016). Mitochondrial ROS produced via reverse electron transport extend animal lifespan. Cell Metab. *23*, 725–734.

Seiferling, D., Szczepanowska, K., Becker, C., Senft, K., Hermans, S., Maiti, P., Konig, T., Kukat, A., and Trifunovic, A. (2016). Loss of CLPP alleviates mitochondrial cardiomyopathy without affecting the mammalian UPRmt. EMBO Rep. *17*, 953–964.

Shao, D., Oka, S., Liu, T., Zhai, P., Ago, T., Sciarretta, S., Li, H., and Sadoshima, J. (2014). A redox-dependent mechanism for regulation of AMPK activation by Thioredoxin1 during energy starvation. Cell Metab. *19*, 232–245.

Shpilka, T., and Haynes, C. (2017). The mitochondrial UPR: mechanisms, physiological functions and implications in ageing. Nat. Rev. Mol. Cell Biol. *19*, 109–120.

Szibor, M., Dhandapani, P., Dufour, E., Holmström, K., Zhuang, Y., Salwig, I., Wittig, I., Heidler, J., Gizatullina, Z., Gainutdinov, T., et al. (2016). Broad AOX expression in a genetically tractable mouse model does not disturb normal physiology. Dis. Model. Mech. *10*, 163–171.

Trifunovic, A., Hansson, A., Wredenberg, A., Rovio, A., Dufour, E., Khvorostov, I., Spelbrink, J., Wibom, R., Jacobs, H., and Larsson, N. (2005). Somatic mtDNA mutations cause aging phenotypes without affecting reactive oxygen species production. Proc. Natl. Acad. Sci. USA *102*, 17993–17998.

Trifunovic, A., Wredenberg, A., Falkenberg, M., Spelbrink, J., Rovio, A., Bruder, C., Bohlooly-Y, M., Gidlöf, S., Oldfors, A., Wibom, R., et al. (2004). Premature ageing in mice expressing defective mitochondrial DNA polymerase. Nature *429*, 417–423.

Viscomi, C., Bottani, E., Civiletto, G., Cerutti, R., Moggio, M., Fagiolari, G., Schon, E., Lamperti, C., and Zeviani, M. (2011). In vivo correction of COX deficiency by activation of the AMPK/PGC-1α axis. Cell Metab. *14*, 80–90.

Viscomi, C., Burlina, A., Dweikat, I., Savoiardo, M., Lamperti, C., Hildebrandt, T., Tiranti, V., and Zeviani, M. (2010). Combined treatment with oral metronidazole and N-acetylcysteine is effective in ethylmalonic encephalopathy. Nat. Med. *16*, 869–871.

Yin, H., Price, F., and Rudnicki, M. (2013). Satellite cells and the muscle stem cell niche. Physiol. Rev. *93*, 23–67.

Yun, J., and Finkel, T. (2014). Mitohormesis. Cell Metab. *19*, 757–766.

CellPress

STAR★METHODS

KEY RESOURCES TABLE

REAGENT or RESOURCE	SOURCE	IDENTIFIER
Antibodies		
Rabbit polyclonal anti-EIF4EBP1	Cell Signaling	Cat#9452; RRID: AB_331692
Goat polyclonal anti-ALDH18A1	Thermo Fisher Scientific	Cat#PA5-19392; PA5-19392
Rabbit polyclonal anti-AMPKα	Cell Signaling	Cat#2532; RRID: AB_330331
Mouse monoclonal anti-AOX	21st Century Biochemicals	Customized
Rabbit polyclonal anti-EIF2α	Cell Signaling	Cat#9722; RRID: AB_2230924
Mouse monoclonal anti-GAPDH	Abcam	Cat#ab8245; RRID: AB_2107448
Rabbit polyclonal anti-GRP75	Abcam	Cat#ab53098; RRID: AB_880311
Mouse monoclonal anti-HSC70 (B-6)	Santa Cruz Biotechnology	Cat#sc-7298; RRID: AB_627761
Rabbit polyclonal anti-HSP60	Abcam	Cat#ab46798; RRID: AB_881444
Rabbit polyclonal anti-LC3B	Novus Biologicals	Cat#NB100-2220; RRID: AB_10003146
Mouse monoclonal anti-MHCI	Developmental Studies Hybridoma Bank	Cat#BA-F8; RRID: AB_10572253
Mouse monoclonal anti-MHCIIa	Developmental Studies Hybridoma Bank	Cat#SC-71; RRID: AB_2147165
Mouse monoclonal anti-MHCIIb	Developmental Studies Hybridoma Bank	Cat#BF-F3; RRID: AB_2266724
Mouse monoclonal anti-SOD2 (2A1)	Abcam	Cat#ab16956; RRID: AB_302569
Rabbit polyclonal anti-MTHFD2	Proteintech	Cat#12270-1-AP; RRID: AB_2147525
Mouse monoclonal anti-MyoD	Novus Biologicals	Cat#NB100-56511; RRID: AB_838603
Rabbit monoclonal anti-NRF2 (D1Z9C) XP	Cell Signaling	Cat#12721; RRID: AB_2715528
Mouse monoclonal anti-p62/SQSTM1 (M01), clone 2C11	Abnova	Cat#H00008878-M01; RRID: AB_437085
Rabbit polyclonal anti-PAX7	Thermo Fisher Scientific	Cat#PA1-117; RRID: AB_2539886
Rabbit polyclonal anti-PGC-1α	Santa Cruz Biotechnology	Cat#sc-13067; RRID: AB_2166218
Rabbit monoclonal anti-phospho-EIF4EBP1 (Thr37/Thr46)	Cell Signaling	Cat#2855; RRID: AB_560835
Rabbit polyclonal anti-phospho-AMPKα (Thr172)	Cell Signaling	Cat#2531; RRID: AB_330330
Rabbit monoclonal anti-phospho-EIF2α (Ser51) (D9G8)	Cell Signaling	Cat#3398; RRID: AB_2096481
Rabbit polyclonal anti-PYCR1	Proteintech	Cat#13108-1-AP; RRID: AB_2174878
Rabbit polyclonal anti-TFAM	Abcam	Cat#ab131607; RRID: AB_11154693
Mouse monoclonal anti-total OXPHOS antibody cocktail	Abcam	Cat#ab110412
Chemicals, Peptides, and Recombinant Proteins		
N-acetyl-L-cysteine	Sigma-Aldrich	Cat#A7250
Hematoxylin solution according to Mayer	Sigma-Aldrich	Cat#51275-1L
Eosin Y	Sigma-Aldrich	Cat#E4009-25G
DAKO Antibody diluent	Agilent Dako	Cat# S3022
Prolong Diamond Antifade with DAPI	Invitrogen	Cat#P36962
TRIzol reagent	Invitrogen	Cat#15596026
DNA-free DNA removal kit	Ambion	Cat#AM1906
Omniscript reverse transcription kit	Qiagen	Cat#205113
TaqMan Assay-on-Demand *Fgf21*	Thermo Fisher	Cat#Mm00840165_g1
TaqMan Assay-on-Demand *Gdf15*	Thermo Fisher	Cat#Mm00442228_m1
TaqMan Assay-on-Demand *Gapdh*	Thermo Fisher	Cat#Mm99999915_g1
TaqMan Assay-on-Demand *Hprt*	Thermo Fisher	Cat#Mm00446968_m1
PowerSYBR Green PCR master mix	Applied Biosystems	Cat#4367659
Trypsin-EDTA (0.5%), no phenol red	Gibco	Cat#15400054
Amplex Red Reagent	Life Technologies	Cat#A12222

(*Continued on next page*)

Continued		
REAGENT or RESOURCE	SOURCE	IDENTIFIER
Horseradish peroxidase	Sigma-Aldrich	Cat#P8250
Superoxide dismutase	Sigma-Aldrich	Cat#S8409
Succinate disodium salt, hexahydrate	Sigma-Aldrich	Cat#S2378
L-Glutamic acid, monosodium salt hydrate	Sigma-Aldrich	Cat#G1626
L-Malic acid	Sigma-Aldrich	Cat#M1000
Pyruvic acid, sodium salt	Sigma-Aldrich	Cat#P2256
ADP	Sigma-Aldrich	Cat#A5285
KCN	Fluka	Cat#60178
Oligomycin	Sigma-Aldrich	Cat#O4876
Rotenone	Sigma-Aldrich	Cat#R8875
Safranin	Sigma-Aldrich	Cat#S2255
Cytochrome c	Sigma-Aldrich	Cat#C7752
DTNB	Sigma-Aldrich	Cat#D8130
Acetyl-Coenzyme A	Sigma-Aldrich	Cat#A2181
Oxaloacetate	Sigma-Aldrich	Cat#O4126
Critical Commercial Assays		
Aconitase activity assay kit	Sigma-Aldrich	Cat#MAK051
ATP determination kit	Molecular Probes	Cat#A22066
Experimental Models: Organisms/Strains		
Mouse: *COX15*$^{sm/sm}$ (KO): Tg/ACTA/BL6	Viscomi et al., 2011	N/A
Mouse: *AOX*: Tg/CAG/BL6	Szibor et al., 2016	N/A
Oligonucleotides		
See Table S1 for primer sequences	This paper	N/A
Software and Algorithms		
GraphPad Prism 7 for Mac OS X	GraphPad Software	Version 7.0d
Fiji	Schindelin et al., 2012	RRID: SCR_002285
Zen Pro	Carl Zeiss Ltd.	14.0.9.201
Fusion	Andor Technologies	1.5.0.7
Imaris	Bitplane	9.1.2
Other		
Pre-cast NuPAGE 4%–12% Bis-Tris gels	Invitrogen	Cat#NP0322BOX

CONTACT FOR REAGENT AND RESOURCE SHARING

More information and requests for resources and reagents should be directed to and will be fulfilled by the Lead Contact, Carlo Viscomi (cvf23@mrc-mbu.cam.ac.uk).

EXPERIMENTAL MODEL AND SUBJECT DETAILS

All procedures were conducted under the UK Animals (Scientific Procedures) Act, 1986, approved by Home Office license (PPL: 70/7538) and local ethical review. The animals were maintained on a C57BL/6-129Sv mixed background at 19-21°C in a temperature- and humidity-controlled animal-care facility, with a 12 hr light/dark cycle and free access to water and food (R105, Safe Diets, Augy, France), and were sacrificed by cervical dislocation. All the experiments were performed using 8-week-old mice. Both males and females were used in the experiments as no gender-related differences were observed.

AOX mice were knocked-in in the ROSA26 locus and under the ubiquitous, early onset CMV immediate enhancer/chicken beta actin (CAG) promoter (Szibor et al., 2016).

Cox15$^{sm/sm}$ mice were supplemented with N-acetyl-L-cysteine (Sigma-Aldrich, A7250) in the drinking water (1%, pH 7.0), which was changed twice per week. Animals were checked and weighed weekly and any that lost 20% of their pre-study weight or showed clinical signs reaching the moderate end point were culled.

Total movement of mice during night was counted using the Comprehensive Lab Animal Monitoring System (CLAMS, Columbus Instruments).

METHOD DETAILS

Histochemistry, Immunofluorescence and Imaging

For histochemical analysis, skeletal muscles were frozen in isopentane pre-cooled with liquid nitrogen. 8 μm thick sections were stained for COX and SDH activity. For SDH analysis six images per sample were acquired randomly in places of mixed intensity fibers keeping the same light intensity and exposure settings using an Axio Observer Z1 with ApoTome 2 (Carl Zeiss Ltd.), composed by a Zeiss 10x ApoPlan objective and an AxioCam ICc1 camera. Images were acquired using Zen Pro software and analysed with Fiji (% of total area) (Schindelin et al., 2012) keeping the same threshold for all samples.

Analysis of cross sectional area and centralized nuclei was performed on hematoxylin and eosin (H & E)-stained sections using Fiji on four samples/genotype (600 fibers/sample).

For immunofluorescence, slides were fixed in 4% PFA for 10 min, washed in PBS and then incubated in 0.2% triton in PBS for 15 min. After washing in PBS, sections were incubated in blocking solution (5% normal goat serum, 2% BSA, 1:40 M.O.M. blocking reagent in PBS) for 1 h at RT and then incubated with primary antibodies diluted in DAKO Antibody diluent with background reducing components for 1 h at RT. Slides were washed in PBS for 15 min and incubated for 1 h at RT with Alexa Fluor secondary antibodies (1:300 in DAKO Antibody diluent with background reducing components). Sections were washed in PBS and mounted with Prolong Diamond Antifade with DAPI (Invitrogen).

For PAX7/MYOD analysis five images were acquired randomly for each slide using a Dragonfly Spinning Disk imaging system (Andor Technologies Ltd.), composed by a Nikon Ti-E microscope, Nikon 20x ApoPlan objective and an Andor Ixon EMCCD camera. The z-stacks were acquired using Fusion software (Andor Technologies) and the 3D images analysed using Imaris software (Bitplane) creating spots surfaces for each channel (MYOD and PAX7) keeping the same conditions for all the samples. A surface was created using the autofluorescence of the tissue to measure the area and the number of positive nuclei was normalized per area.

Fiber typing analysis was calculated from entire muscle sections using a Tile image acquired using the Dragonfly Spinning Disk as previously described for MYOD/PAX7. The morphometric analysis was performed using Imaris software creating surfaces for each channel.

No blinding was used for any of the histological/immunofluorescence analysis. No samples were excluded from analysis.

Analysis of Mitochondrial Enzyme Activities

Skeletal muscle samples were snap-frozen in liquid nitrogen and homogenized in 10 mM phosphate buffer (pH 7.4). The spectrophotometric activities of cIV and citrate synthase (CS) were measured as described (Bugiani et al., 2004). In brief, complex IV activity was measured by following reduction of cytochrome c at 550 nm at 37°C. 10 μg mitochondria were incubated in a mix containing 10 mM KH_2PO_4, pH 7.2 and between 5 and 50 μ M cytochrome c.

Citrate synthase activity was measured at 30°C by incubating 10 μg mitochondria in a reaction mixture containing 125 mM Tris–HCl, 100 μ M DTNB (5,5′-dithiobis(2-nitrobenzoic acid)) and 300 μ M acetyl coenzyme A. The reaction was initiated by the addition of 500 μ M oxaloacetate, and DTNB reduction at 412 nm measured for 2 min.

Analysis of Body Composition

Nuclear magnetic resonance (NMR) was employed to determine the body fat content of live animals using the NMR Analyzer Minispec mq7.5 (Bruker Optik, Ettlingen, Germany).

Real-Time Quantitative Polymerase Chain Reaction

RNA was isolated from skeletal muscles using TRIzol reagent (Invitrogen, 15596026) according to manufacturer's instructions, treated with DNAse (DNA-free Kit, Ambion) and subsequently reversely transcribed with the Omniscript reverse transcription kit (Qiagen, 205113). *Fgf21* (Mm00840165_g1), *Gdf15* (Mm00442228_m1), *Gapdh* (Mm99999915_g1) and *Hprt* (Mm00446968_m1) probes were obtained from TaqMan Assay-on-Demand kits (Applied Biosystems).

All the other transcripts, including mtDNA content, were analyzed by SYBR Green real-time PCR. Samples were adjusted for total RNA content by *Hprt*, *Gapdh* or in the case of mtDNA content, *RNaseP*. Relative expression of mRNAs was determined using a comparative method ($2^{-\Delta\Delta CT}$). Primer sequences are provided in Table S1.

Western Blot Analysis

Proteins, extracted from homogenized skeletal muscle samples, were separated by denaturing NuPAGE 4%–12% Bis-Tris gels. The primary antibodies were used in dilutions recommended by the suppliers. The list of antibodies is included in the Key Resources Table.

Mitochondria Isolation and H_2O_2 Production

Mitochondria were isolated from skeletal muscle as described (Frezza et al., 2007). Briefly, small pieces of skeletal muscles were incubated in ice-cold PBS/10 mM EDTA and 0.05% trypsin for 30 minutes on ice. After centrifugation at 200 g for 5 min at 4°C,

the pellet was resuspended in IBM1 buffer (67 mM Sucrose, 50 mM KCl, 50 mM Tris-HCl pH 8, 10 mM EDTA, 0,2% BSA free from fatty acids, pH 7.4). The pieces were homogenized by hand, centrifuged at 700 g for 10 minutes at 4°C. The supernatant was further centrifuged at 8000 g for 10 minutes at 4°C to pellet mitochondria, which was resuspended in IBM2 buffer (250 mM sucrose, 0.3 mM EGTA-Tris, 10 mM Tris-HCl, pH 7.4).

H_2O_2 production rate was measured at 37°C using 130 μg of mitochondria diluted in 2 ml of mitochondrial respiration buffer (120 mM sucrose, 50 mM KCl, 20 mM Tris–HCl, 4 mM KH_2PO_4, 2 mM $MgCl_2$, 1 mM EGTA, 1 mg/ml fatty-acid-free BSA, pH 7.2) in an Oxygraph-2k using O2k-Fluo LED2-Module (OROBOROS INSTRUMENTS, Innsbruck, Austria). The rationale behind the method is monitoring the oxidation of the fluorogenic indicator Amplex Red Reagent (Life Technologies, A12222) in the presence of horseradish peroxidase (Sigma-Aldrich, P8250) and superoxide dismutase (Sigma-Aldrich, S8409) (Krumschnabel et al., 2015). The final concentrations of Amplex Red, horseradish peroxidase and superoxide dismutase in the incubation medium were 10 μM, 4 U/ml and 5 U/ml, respectively. H_2O_2 production rate measurements were initiated by succinate (final concentration 10 mM) addition as a substrate for cII. In a separate experiment, a standard curve was obtained by adding known amounts of H_2O_2 to the assay medium in the presence of the reactants. The H_2O_2 production rate was determined from the slope of a plot of the fluorogenic indicator versus time.

For (cyanide-resistant) respiration, after addition of cI-linked or cII-linked substrates, including 1 mM ADP, 1 mM KCN or 2.5 μg/ml oligomycin was added into the Oxygraph-2k chambers.

Mitochondrial Aconitase Activity

Mitochondrial aconitase 2 activity was measured by Aconitase Activity Assay Kit (Sigma-Aldrich, MAK051) according to the manufacturer's protocol.

QH_2/Q Measurements

In order to measure the proportion of both reduced and oxidized CoQ, a rapid lipid extraction was performed. Briefly, 10 to 15 mg of muscle tissue was homogenized with a manual micropestle in 20 mM potassium phosphate buffer pH 7.5, and debris were discarded by maximum speed centrifugation, at 4°C. 100 ml of sample was mixed by vortex with 330 μl of n-propanol in the presence of 0.5 mM β-mercaptoethanol. Sample debris were discarded after spin at maximum speed and clean lipid extracts were immediately injected into a reverse-phase Beckmann 166 HPLC system, equipped with a C18 column (5 μm, 150 x 4.6 mm) and column oven set up at 40°C. Mobile phase, flow rate and gradient settings were as described previously (Rodríguez-Aguilera et al., 2017). CoQ detection was performed by a Coulochem III ESA electrochemical detector linked to the HPLC system and conditioning cell was set up before injection valve in order to maintain the oxidation state of the sample analytes.

Measurement of ATP Synthesis Flux and ATP Content

ATP production rate was assessed as described (Mourier et al., 2015). Briefly, isolated skeletal muscle mitochondria (65 μg/ml) were suspended in the mitochondrial respiration buffer (see "Measurement of H_2O_2 production"). After addition of ADP (1 mM), succinate (2 mM) and rotenone (10 nM) or addition of ADP (1 mM), pyruvate (10 mM), glutamate (5 mM) and malate (5 mM), aliquots were collected every 20 seconds and precipitated in 7% $HClO_4$/25 mM EDTA, centrifuged at 16,000 g_{max} for 10 minutes, and then neutralized with 2 M KOH and 0.3 M MOPS. The ATP content in these samples was determined with ATP determination kit (Molecular Probes, A22066) according to the manufacturer's instructions. In a parallel experiment, oligomycin (2.5 μg/ml protein) was added to the mitochondrial suspension to determine the nonoxidative ATP synthesis rate.

ATP content in frozen skeletal muscle samples was measured as described (Fernandez-Marcos et al., 2012) using the same kit as above. Briefly, tissues were weighed and lysed in 600 μl of 6% (v/v) perchloric acid, and centrifuged at 4,000 g_{max} for 10 minutes at 4°C. 500 μl of the supernatant were neutralized with 200 μl of 2.5 M KOH, and precipitate was removed by centrifugation at 4,000 g_{max} for 5 minutes at 4°C. Luciferase activity was measured with a lag time of 1 second and an integration time of 3 seconds using a GloMax 96 Microplate Luminometer (Promega).

Mitochondrial Membrane Potential Measurement

Mitochondrial membrane potential was assessed as described (Chowdhury et al., 2015). In brief, Oxygraph-2k was used with O2k-Fluorescence LED2 module equipped with filter sets for safranin (excitation at 495 nm and emission at 587 nm). Safranin (Sigma-Aldrich, S2255) dissolved in distilled water up to a final concentration of 2.5 μM. Isolated skeletal muscle mitochondria (65 μg/ml) were suspended in the mitochondrial respiration buffer (see "Measurement of H_2O_2 production") including 2.5 μM safranin. The membrane potential was tracked live with the addition of ADP (2.5 mM), pyruvate (5 mM), glutamate (10 mM) and malate (5 mM) or ADP (2.5 mM), succinate (10 mM) and rotenone (0.5 μM). In a separate experiment, a standard curve was obtained by adding known amounts of safranin to the assay medium in the presence of mitochondria. The signal was then normalized (0–1) to detect changes after addition of substrates (2.5 μM safranin set as 1).

QUANTIFICATION AND STATISTICAL ANALYSIS

Fiji software (Schindelin et al., 2012) was used for quantification. Statistical analyses were performed using GraphPad Prism 7 for Mac OS X (GraphPad Software; La Jolla, CA, USA).

Unpaired Student's t-test was used for pairwise comparisons of independent experimental groups. Kaplan–Meier distribution and one sample t-test were used for survival analysis. No particular method was used to determine whether the data met assumptions of the statistical approach. Grubb's test was used to determine outliers. Error bars represent standard error of the mean (S.E.M.).

Cell Metabolism, Volume *28*

Supplemental Information

Perturbed Redox Signaling

Exacerbates a Mitochondrial Myopathy

Sukru Anil Dogan, Raffaele Cerutti, Cristiane Benincá, Gloria Brea-Calvo, Howard Trevor Jacobs, Massimo Zeviani, Marten Szibor, and Carlo Viscomi

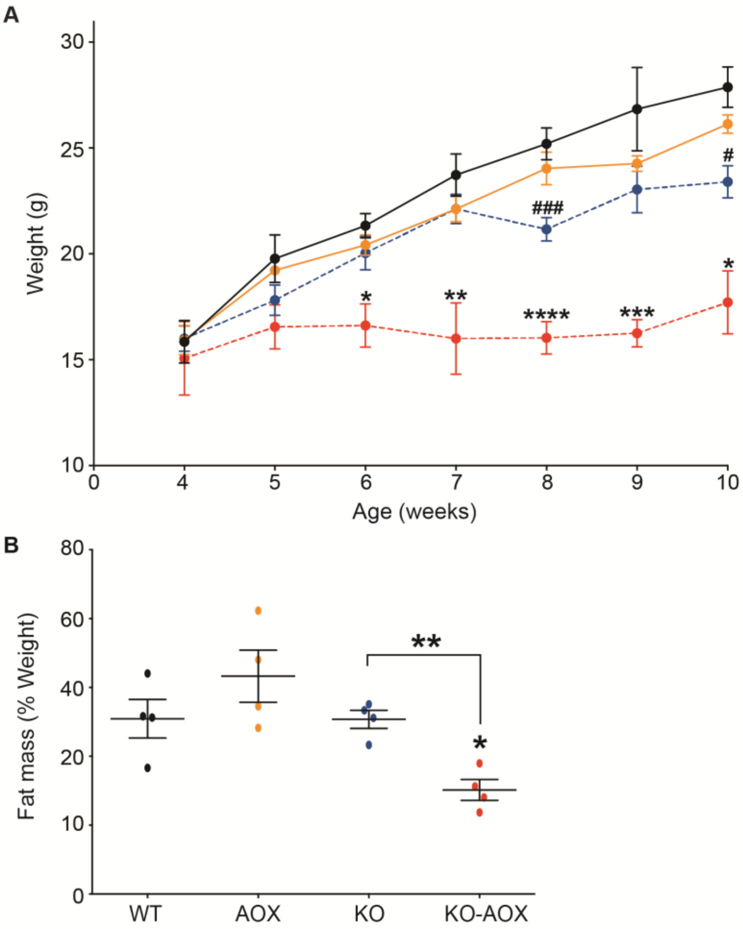

A
30
25
20
15
10
Weight (g)
0
4
5
6
7
8
9
10
Age (weeks)
###
#
*
*
**
****
***
B
80
60
40
20
10
0
Fat mass (% Weight)
**
*
WT
AOX
KO
KO-AOX

Figure S1. Related to Figure 1. KO-AOX is phenotypically worse than KO and control mice.

(A) Weight curve for male WT, AOX, KO and KO-AOX mice. Asterisks are used to compare the weight between KO and KO-AOX; the hash keys between WT and KO (n = 13-19). **(B)** Percentage of body fat in 8-week-old animals measured by NMR (n = 4).

Bars represent mean ± S.E.M. Asterisks and hash keys over the bars indicate statistical significance vs. WT; over the brackets among indicated groups. (*p, #p $\leq$ 0.05; **p $\leq$ 0.01; ***p, ###p $\leq$ 0.001; ****p < 0.0001; unpaired Student's *t*-test).

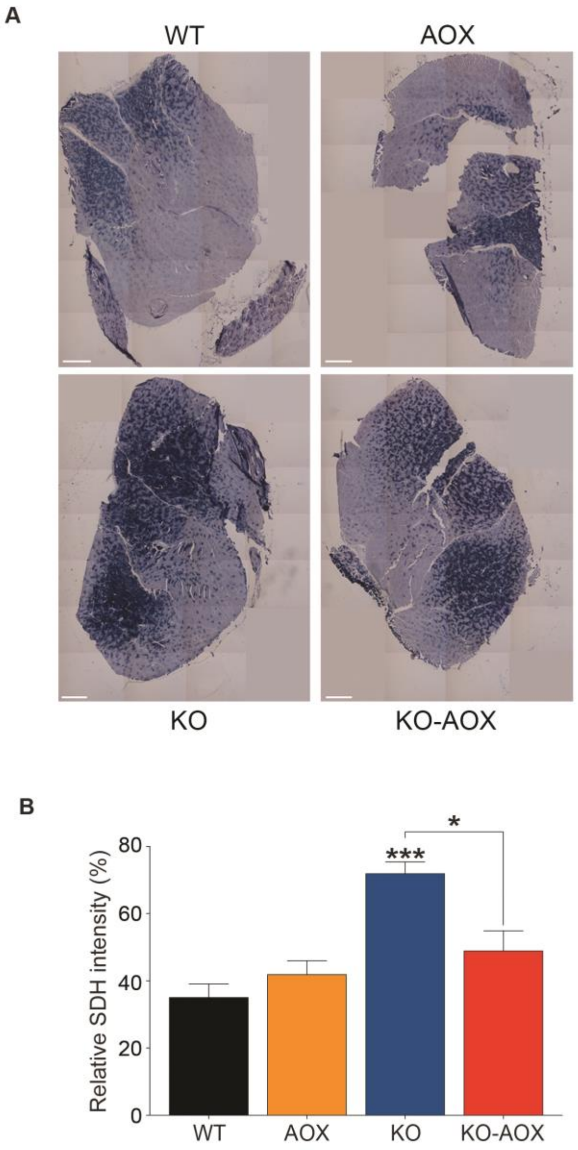

A
WT
AOX
KO
KO-AOX
B
Relative SDH intensity (%)
80
60
40
20
0
***
*
WT
AOX
KO
KO-AOX

Figure S2. Related to Figure 2. SDH activity is increased in KO muscles but returns to WT levels in KO-AOX.

(A) Representative gastrocnemius muscle SDH activity micrograph. Whole muscle fiber was stained for SDH activity in 8-week-old WT, AOX, KO and KO-AOX animals (blue). The image represents a randomly chosen image from four samples. Scale bar: 500 µm. **(B)** Quantification of relative SDH intensity (% of total area) (n = 4).

Bars represent mean ± S.E.M. Asterisks over the bars indicate statistical significance vs. WT; over the brackets among indicated groups. (*$p \leq 0.05$; ***$p \leq 0.001$; unpaired Student's *t*-test).

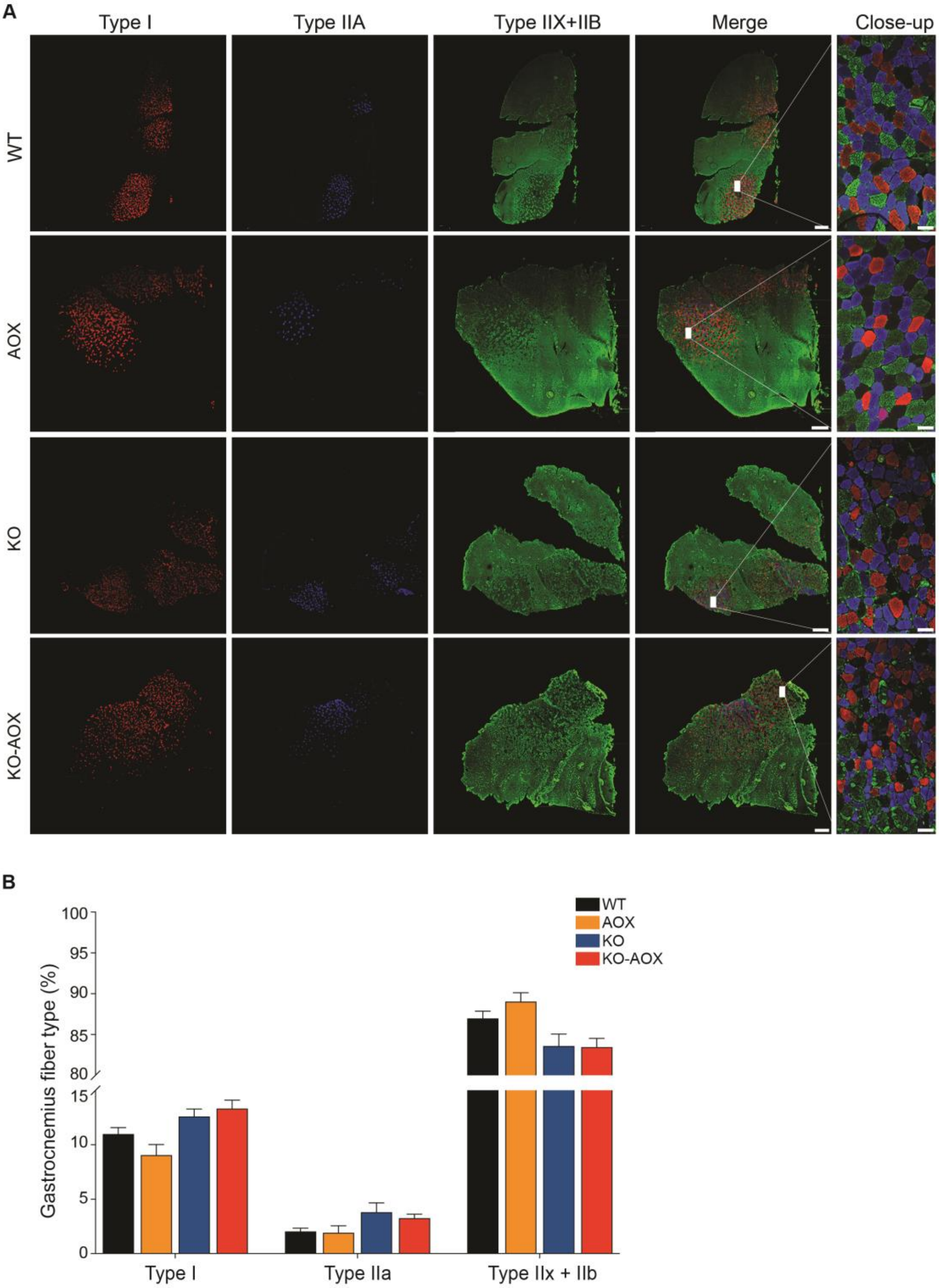
A
Type I
Type IIA
Type IIX+IIB
Merge
Close-up
WT
AOX
KO
KO-AOX
B
WT
AOX
KO
KO-AOX
Gastrocnemius fiber type (%)
100
95
90
85
80
15
10
5
0
Type I
Type IIa
Type IIx + IIb

Figure S3. Related to Figure 3. No differences were observed in the distribution of muscle fiber types among WT, AOX, KO and KO-AOX animals.

(A) Sections of 8-week-old gastrocnemius muscle were immunostained for the different MHCI, MHCIIA and MHCIIB myosins. Type I (Red), type IIA (Blue), type IIB (Green) and type IIX (unstained) fibers are shown in a whole tissue 3D montage. Scale bars: WT and KO 500 μm, AOX 400 μm and KO-AOX 300 μm. White rectangle highlights the zoomed area (scale bar: 50 μm). **(B)** Quantification of percentage of each fiber per whole tissue section (n = 4).

Bars represent mean ± S.E.M.

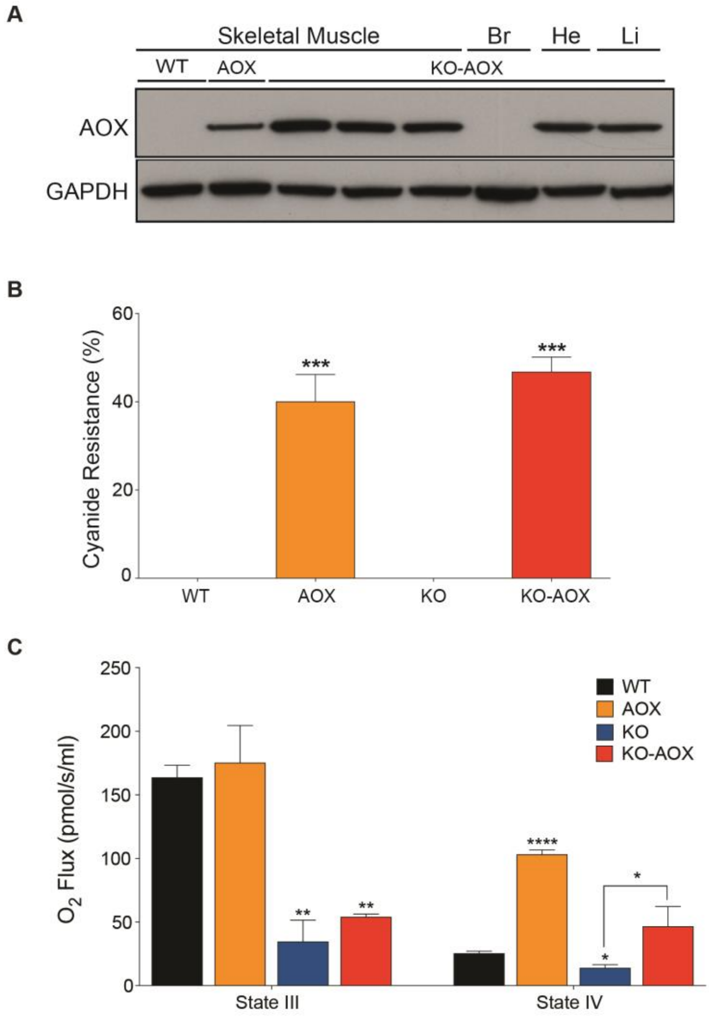

A
Skeletal Muscle
Br
He
Li
WT
AOX
KO-AOX
AOX
GAPDH
B
Cyanide Resistance (%)
60
40
20
0
***
***
WT
AOX
KO
KO-AOX
C
O2 Flux (pmol/s/ml)
250
200
150
100
50
0
WT
AOX
KO
KO-AOX
**
**
****
*
*
State III
State IV

Figure S4. Related to Figure 4. AOX is expressed and active in KO-AOX.

(A) Western blot analysis of AOX in skeletal muscle, brain (Br), heart (He) and liver (Li) of WT, AOX and KO-AOX mice. GAPDH is used as loading control. **(B)** Cyanide resistance in AOX and KO-AOX mice measured by percent of State III (ADP-stimulated) respiration (n=4). **(C)** Succinate-driven State III (ADP-stimulated) and State IV (oligomycin-sensitive) oxygen consumption rates in isolated skeletal muscle mitochondria (n = 4).

All experiments were done on 8-week-old animals. Bars represent mean ± S.E.M. Asterisks directly on top indicate level of statistical significance compared to WT (*p ≤ 0.05; **p ≤ 0.01; ***p ≤ 0.001; ****p < 0.0001; unpaired Student's *t*-test).

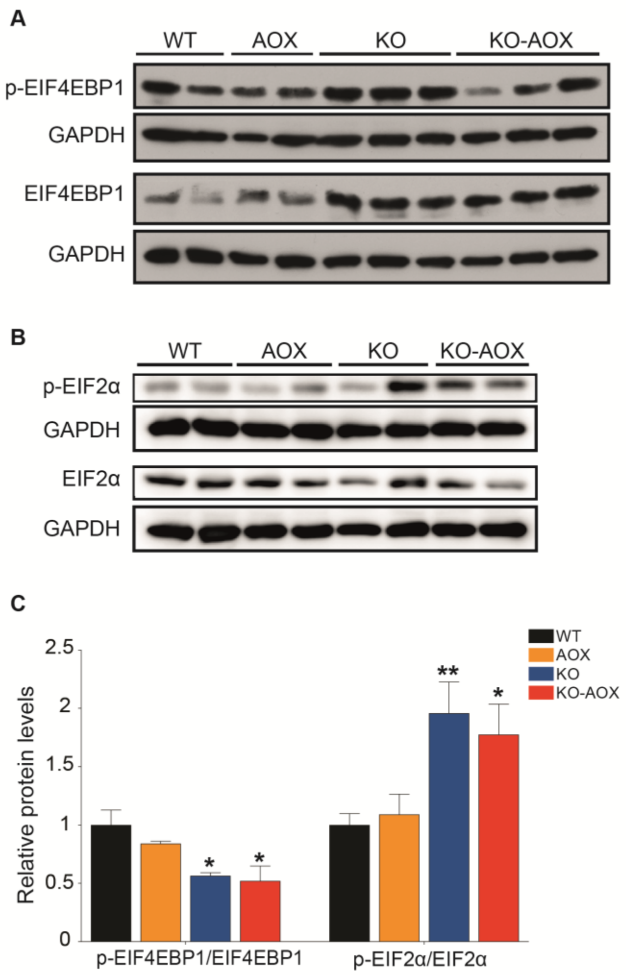

A
WT
AOX
KO
KO-AOX
p-EIF4EBP1
GAPDH
EIF4EBP1
GAPDH
B
WT
AOX
KO
KO-AOX
p-EIF2α
GAPDH
EIF2α
GAPDH
C
WT
AOX
KO
KO-AOX
Relative protein levels
2.5
2
1.5
1
0.5
0
*
*
**
*
p-EIF4EBP1/EIF4EBP1
p-EIF2α/EIF2α

Figure S5. Related to Figure 6. Both KO and KO-AOX show increased levels of EIF-2alpha (EIF2α) and decreased mTOR signaling.

Western blots of **(A)** phosphorylated- and total EIF4EBP1; and **(B)** phosphorylated- and total EIF2α. **(C)** Quantification (n = 5) of aforementioned proteins in WT, AOX, KO and KO-AOX mice.

Western blots were performed on skeletal muscle homogenates of 8-week-old mice. GAPDH is used as loading control. Bars represent mean ± S.E.M. Asterisks over the bars indicate statistical significance vs. WT; over the brackets among indicated groups. (*$p \leq 0.05$; **$p \leq 0.01$; unpaired Student's *t*-test).

Table S1. Related to Star Methods. qPCR primer sequences.

	Forward 5' - 3'	Reverse 5' - 3'
Afg3l2	GTTGATGGGCAATACGTCTGG	GACCCGGTTCTCCCCTTCT
Aldh18a1	AATCAGGGCCGAGAGATGATG	GGCCTCTAAGACCGGAATTGC
Atf3	CCAGAATAAACACCTCTGCCATCG	CTTCAGCTCAGCA TTCACACTCTC
Atf4	GCAAGGAGGATGCCTTTTC	GTTTCCAGGTCATCCATTCG
Atf5	CCTTGCCCTTGCCCACCTTTGAC	CCAGAGGAGGAGGCTGCTGT
Atf6	TCGCCTTTTAGTCCGGTTCTT	GGCTCCATAGGTCTGACTCC
Catalase	TGGCACACTTTGACAGAGAGC	CCTTTGCCTTGGAGTATCTGG
Chop	CTGGAAGCCTGGTATGAGGAT	CAGGGTCAAGAGTAGTGAAGGT
CoxI	TGCTAGCCGCAGGCATTACT	CGGGATCAAAGAAAGTTGTGTTT
CoxII	CAGGCCGACTAAATCAAGCAA	GAGCATTGGCCATAGAATAATCCT
CoxVa	TCATCCAGGAACTTAGACCAACT	AGTCCTTAGGAAGCCCATCG
Gpx1	CCACCGTGTATGCCTTCTCC	AGAGAGACGCGACATTCTCAAT
Hprt	TCCTCCTCAGACCGCTTTT	CCTGGTTCATCATCGCTAAT
mtHsp70	ATGGCTGGAATGGCCTTAGC	ACCCAAATCAATACCAACCACTG
Ndufs8	CTTCGGCTTTGTGGCTTTCATGGT	AAAGCCCATCAAGCCTCCTCAGAT
Nfe2l2	TCCATTCCCGAATTACAGTGTCT	GCCCACTTCTTTTTCCAGCG
Pycr1	ATGAGCGTAGGCTTCATCGG	GTGTCAGGTTCACCCCTATCT
RNaseP	GCCTACACTGGAGTCCGTGCTACT	CTGACCACACACGAGCTGGTAGAA
Sdhb	GACGTCAGGAGCCAAAATGG	CTCGACAGGCCTGAAACTGC
Sod1	CAAGCGGTGAACCAGTTGTG	TGAGGTCCTGCACTGGTAC
Sod2	GCCTGCACTGAAGTTCAATG	ATCTGTAAGCGACCTTGCTC
Uqcrfs1	ATCCCTGAAGGGAAGAACATGGCT	TGCAGCTTCCTGGTCAATCTCCTT
